# German energy transition (Energiewende) and what politicians can learn for environmental and climate policy

**DOI:** 10.1007/s10098-020-01939-3

**Published:** 2020-10-04

**Authors:** Rudolf Rechsteiner

**Affiliations:** Eidgenossische Technische Hochschule Zurich, Zurich, Switzerland

**Keywords:** Energy policy, Pigou tax, clean flying, German environmental policy, dynamic efficiency, Swiss environmental policy

## Abstract

The German Energiewende (energy transition) started with price guarantees for avoidance activities and later turned to premiums and tenders. Dynamic efficiency was a core concept of this environmental policy. Out of multiple technologies wind and solar power—which were considered too expensive at the time—turned out to be cheaper than the use of oil, coal, gas or nuclear energy for power generation, even without considering externalities. The German minimum price policy opened doors in a competitive way, creating millions of new generators and increasing the number of market participants in the power sector. The fact that these new generators are distributed, non-synchronous and weather-dependent has caused contentious discussions and specific challenges. This paper discusses these aspects in detail and outlines its impacts. It also describes Swiss regulations that successfully launched avoidance technologies or services and asks why exactly Pigou's neoclassical economic approach to the internalization of damage costs (externalities) has rarely worked in policy reality, while sector-specific innovations based on small surcharges have been more successful. Based on the model of feed-in tariffs, a concept for the introduction of low-carbon air traffic is briefly outlined.

**Graphic Abstract:**

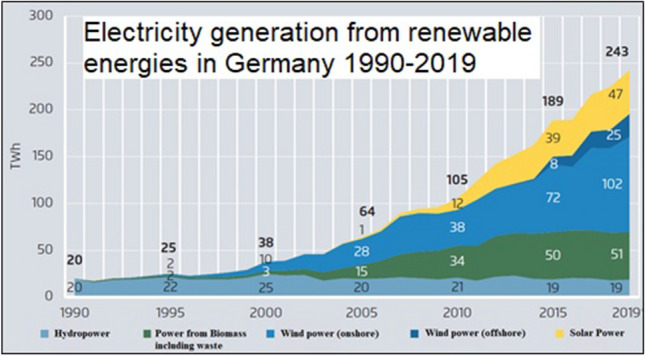

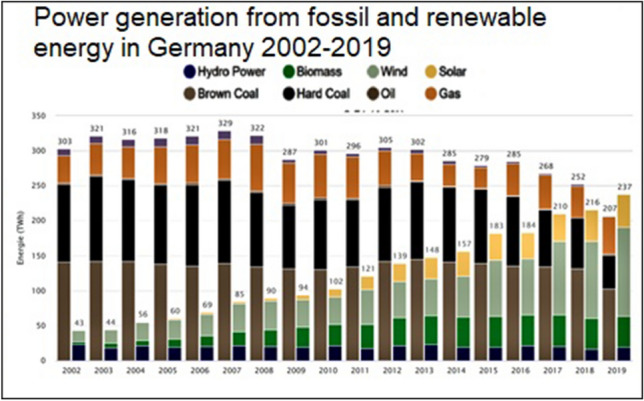

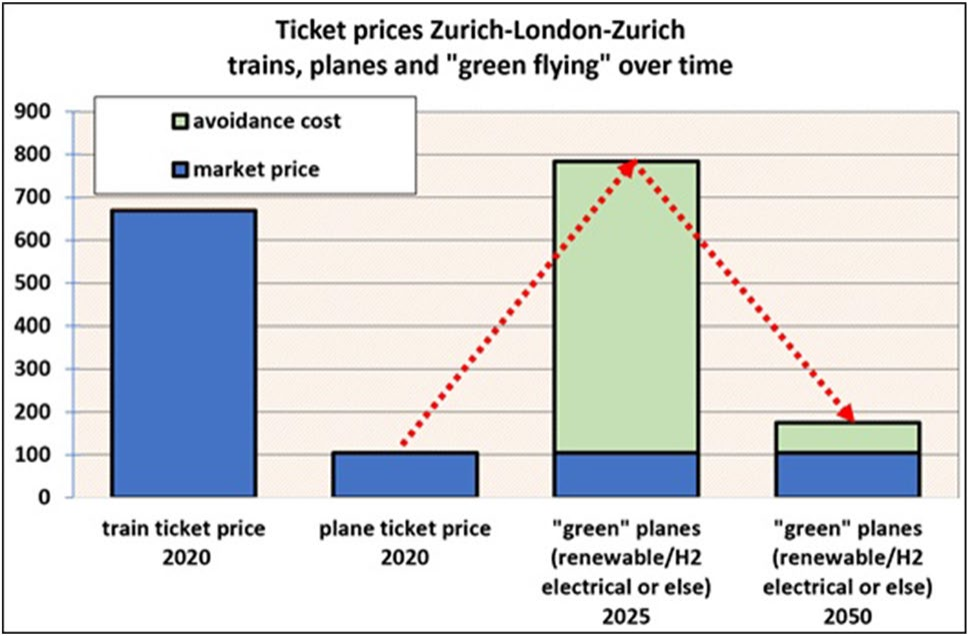

## Introduction

The continuous increase in greenhouse gases in the atmosphere is a cause for concern to many people engaged in environmental policy. The situation is similar to the 1980s when young people opposing the nuclear arms race and nuclear power plants faced an uphill battle, even after the Three Mile Island and Chernobyl accidents caused the spread of radiation and radioactive fallout.

The following essay is written by an economist who, as a member of national and cantonal Parliaments for 30 years (1988–2018), has tried to put economic instruments at the heart of environmental policy. It addresses policy strategies in Germany and Switzerland that decisively launched the development of avoidance strategies, industries and processes.

The German Energiewende (energy transition) was an exemplary model of a new policy approach and caused a fierce reduction in the cost of electricity generation by renewable energy sources, as explained in “The German Energiewende (energy transition): a successful environmental policy” section.

Due to intermittency and low marginal prices of renewables, flexibility, sector coupling and market restructuring were logical complements to the energy transition and changed energy supplies in the traffic, heating and industry sectors. These structural changes will produce winners, losers and laggards. In this context, some challenges to and allegations against transforming the energy sector are discussed in “New (old) arguments and real performance” section.

The price drop of renewable energy output is disrupting national and international energy systems and trade and, ultimately, can reduce the use of fossil fuels worldwide. The main trends in this restructuring are explained in “The relevance of renewables: restructuring and expansion” section.

“Swiss policy attempts promoting avoidance” section discusses examples from Switzerland that illustrate potential policy tools and institutional approaches that are not based on the standard solution of a Pigou levy. They offer valuable insights into changes in the modal split of traffic and in the circular management of waste.

The German energy transition is not yet complete and must extend beyond electricity supply. But already it may have enabled a more thorough advancement of price and volume regulations such as those in the European Emissions Trading scheme or in the revision of the Swiss CO_2_ levies that deliver incentives for a change toward low-carbon technologies. Supporters of economic instruments often think that it is sufficient to internalize externalities through prices, after which the "invisible hand of the market" will do everything so that externalities disappear to a reasonable extent. However, reality is more complicated. Rather than taxes, avoidance strategies with coordinated efforts should be put at the center of any regulation, as discussed in “Priceless environment” section.

Finally, using the example of air traffic, we consider which findings can be transferred to other policy areas in “Final thoughts on clean flying” section.

## The German Energiewende (energy transition): a successful environmental policy

**Fig. 1:**
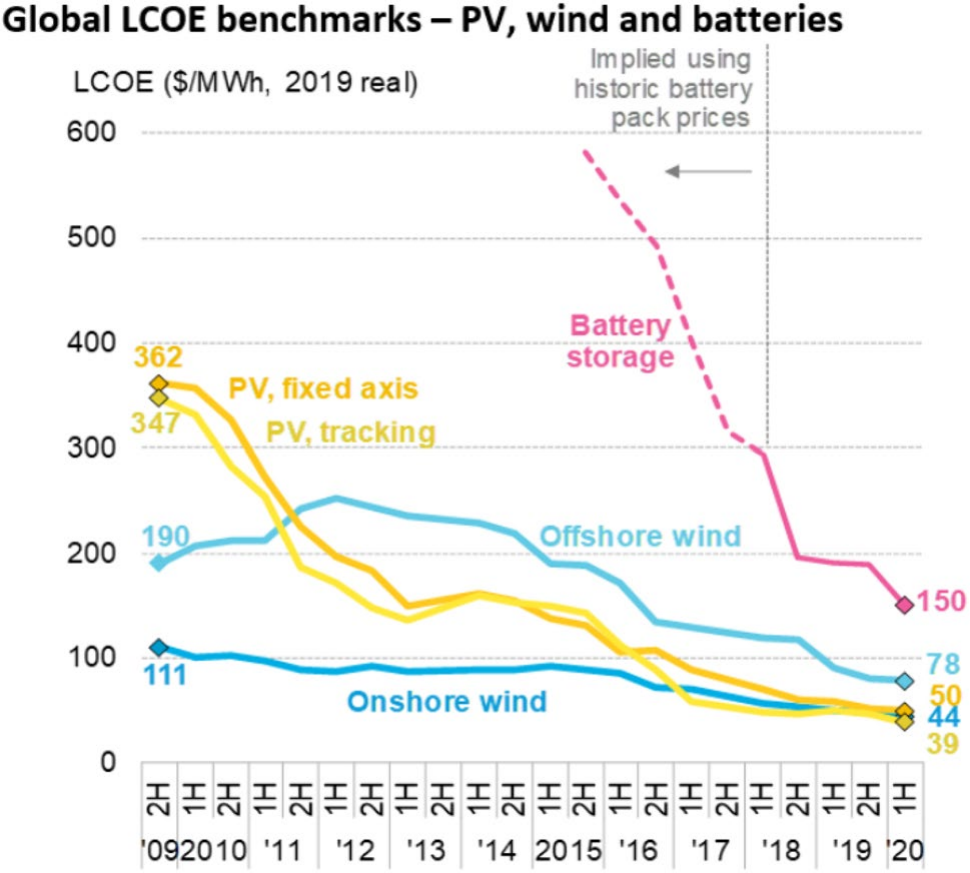
Production costs of electricity from new renewable energies and batteries (BNEF 2020)

From 1990 to 2020, solar photovoltaic (PV)^2^ and wind power generation changed from being two of the most expensive technologies to being the least expensive energy sources worldwide (Fig. 1). This achievement, which had disruptive consequences, was the result of a courageous environmental policy initiated in the German Parliament and assisted by other renewable energy hot spots around the globe (Irena 2020). The affordability of renewable energy will shape the twenty-first century. But the transition is far from complete, and challenges persist, while market shares of new technologies continue to grow.

The raw material for the first industrially produced solar cells was silicon waste from the semiconductor industry. The efficiency of the best solar cells in the laboratory rose from a few percent (1953) to over 47 percent (NREL 2020; Fraunhofer 2020a, b). Today, the best standard module efficiencies range from 20 to 23 percent (Energysage 2020). With solar irradiation of 1000 kWh per m^2^ typical for Central Europe, they allow for 150–200 kWh of power generation per year. Inverter efficiency has increased from approximately 80 percent to over 98%. The balance of system costs including, for example, costs for metal frames, arrays, cabling, planning, installation, storage (batteries, electrolysis, heat) and the required area per kWh have been reduced as well.

Initially, the focus of PV was on off-grid electrification. New applications for self-consumption, for grid-connected customers and for grid injection expanded over time. New applications such as power for heat pumps and storage, electric vehicles and batteries created additional, flexible possibilities for utilization. This mode of expansion is inherent to new technologies, but there can be delayed take-offs ("hysteresis") when incumbent companies overstretch old technologies because they do not know or trust new ones or because they fear a loss of market share (Arthur 2011).

1998, the deregulation of the power and gas markets began. Liberalization was the overarching condition for the expansion of new technologies. The unbundling of grids and power plants and the creation of competitive power markets with—to some extent—a level playing field were merits of the European Union’s regulatory framework (Schiffer et al. 2018).

Today, electricity from PV systems primarily serves four different markets: (1) captive locations without grid connection, (2) solar farms for wholesale power trading, (3) grid-connected systems for self-consumption and (4) micro-grids for industrial, residential and commercial complexes, ships, villages or entire city districts, including storage and black start facilities.

Together with old and new technologies such as heat pumps, heat storage, electric vehicles, battery storage and electrolysis, solar and wind power form a disruptive family. Their combined abilities go far beyond what each technology can achieve on its own. Today, solar-wind-hybrid concepts with storage can offer a security of supply equal to that provided by conventional energy services, and at a lower cost (Vedel 2020).

With new global installations of 115 GW PV and 60 GW wind power in 2019, these technologies have become less dependent on the whims of local government coalitions (IEA-PVPS 2020; GWEC 2020). The balance of forces within the electricity sector has changed in many countries or states. Even old, depreciated coal, gas or nuclear power plants can be undercut by new solar and wind farms’ low costs, expenses for storage, and included frequency control (BNEF 2020).

Self-consumption of solar power from rooftops can reduce costs for commercial establishments and households. Business-friendly governments can no longer ignore this. Photovoltaics and wind power are displacing gas, coal and nuclear power plants, and recently, they have started to compete with petrol, diesel and heating oil in the transport and heating sectors.

Electricity-based technologies can be far more efficient than those powered by thermal engines. An electric motor uses energy three to five times more effectively than a combustion engine. Heat pumps up-cycle the power input by a factor of 3 to 4 by compressing air from ambient heat or other low-temperature thermal sources.

Power from renewable energies is mainly produced and used regionally or locally. Where transport distances are reduced, energy losses are lower. Thanks to the ubiquitous availability of the primary renewable energy sources of wind and solar, and due to advances in energy storage technologies, renewable energy systems can achieve a higher level of resilience than their fossil or nuclear fueled predecessors.

### Learning curves

Competitiveness and affordability were important drivers of market growth. The price per watt of installed PV capacity fell from $100/W in the early 1970s (Perlin 1999) to around $0.5/W (megawatt-sized systems, ground-mounted, Lazard 2019; Pfeiffer et al. 2020). German tenders for solar power resulted in a generation cost of € 0.05/kWh (BnetzA 2020). In California, the "historically lowest average price" stood at $0.0282 per kWh in 2019 and the source was renewable energy (CPUC 2020). In the Middle East, prices were below $0.02/kWh (Bellini 2020). And in India, Solar Energy Corporation of India has concluded its 400 MW “round-the-clock” renewables tender to supply 24-h electricity at $0.038/kWh (Gupta 2020).

The learning curve for solar modules between 1976 and 2019 was 23.5 percent (VDMA 2020), and that of balance of system costs stood at 11 percent (Elshufara et al. 2018). A learning curve of 20 percent means that with each doubling of cumulatively installed capacity, the materials and manufacturing costs fall by 20 percent, which is then reflected in lower prices. Installed PV capacity worldwide doubled seven times between 2000 and 2019 (BP 2020). Prices dropped by more than 80 percent. Even during the winter season, PV can be a viable solution in certain locations—in alpine regions, for example (Häberlin 2012; Rohrer 2019; Rechsteiner et al. 2018; Rechsteiner 2019).

### The case of wind power

**Fig. 2:**
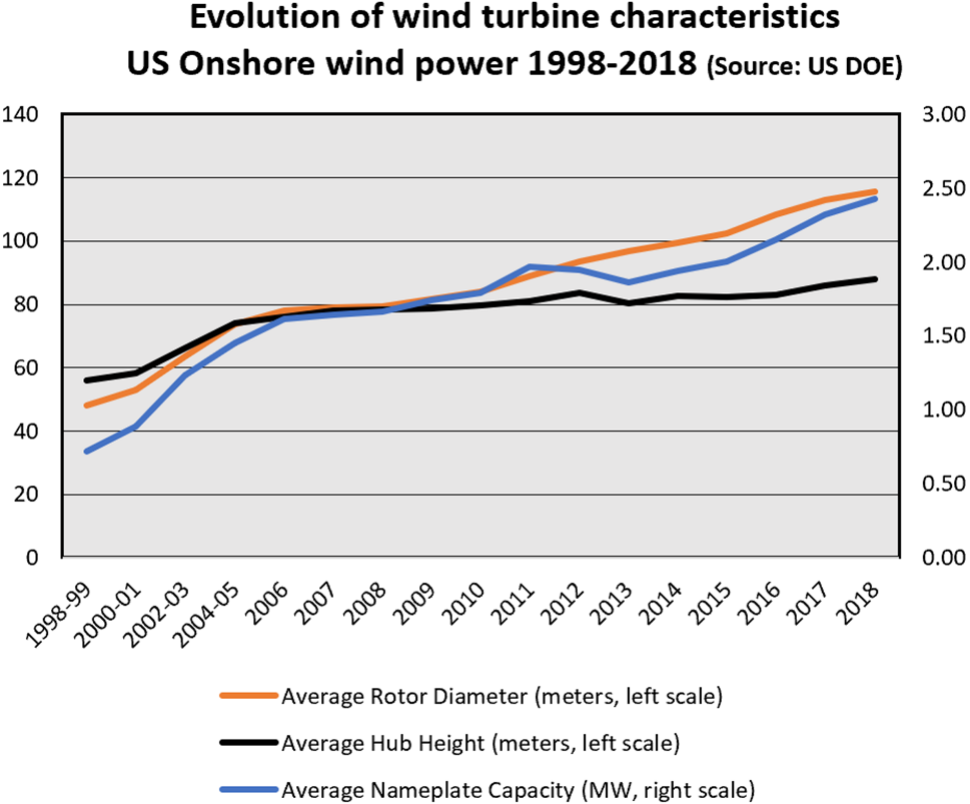
Evolution of US wind turbine characteristics 1998–2018 (US DOE 2019)

Wind power had a similar learning curve, though less steep. Longer blades and new materials had a cost-reducing effect. A tenfold increase in rotor length increased the nominal power ratings by a factor of approximately 100,^1^ while an increase in hub heights opened-up new air layers with more reliable and stronger winds (Fig. 2).

Wind power is more dependent on well-dimensioned power grids than PV. Geographically dispersed usage over large, interconnected areas (EU, USA, China) can balance the intermittency of high and low wind speeds at different locations and can therefore deliver cost-reducing effects.

A strong interest in PV and wind power and grid congestion has led to location diversification. Today, solar power is not only installed on rooftops, but also in open fields, deserts, floating on water surfaces and on the sea, as a provider of shade on farmland (“agrophotovoltaics”), on textiles and on vehicle roofs. For wind power, marine sites have recorded the highest growth rates. Winds are more regular over the sea, and site permits can be managed by central authorities.

### Drivers and extent of the energy transition in Germany

What exactly were the key drivers of these technical, environmental, and economic achievements?

Publications such as "Limits to Growth" (Meadows et al. 1972) led to increased attention to natural capital and scarcities. After the first oil price crisis (1973), many people refused to accept shortages and undesirable spillovers from extractive industries. Environmental movements became active. They opposed foreign dependence on dwindling oil, gas and raw materials, armed conflicts, monopolistic market structures, air or sea pollution, oppression of indigenous peoples, nuclear accidents and radioactive waste, and later, they fought climate change.

A deep rift ran through the midst of society over whether nuclear power was a problem or the solution to the problem. Today, this question has become obsolete because accidents and lack of competitiveness have disqualified the nuclear industry’s pretention as a savior of the climate that is “too cheap to meter” (Strauss 1954).

Historically outstanding was the fact that for an entire generation, opposition to nuclear power created many thousands of small pioneers of wind and solar technologies. These included technicians and small investors in self-consumption or in grid-connected, distributed generation. After 1970, opponents of nuclear power won majorities or strong minorities in many local and national parliaments. Their efforts reduced nuclear risks, and their engagement provided a basis for climate policy.

There was still a long way to go. After World War II, nuclear research dominated the budgets of universities, research institutes and militaries (Fischer 2019). "Atomic ministries" were founded. The risks and failures of commercial applications became apparent with the first meltdown of a commercial reactor at the Three Mile Island nuclear power plant (Pennsylvania, USA) in 1979. Previously, accidents of this kind were presented as impossible or extremely improbable. Afterward, safety requirements were tightened. This made new plants more expensive and curbed commercial interest from utilities operating in competitive power markets (Keystone 2007; Grubler 2010).

The next severe meltdown (Chernobyl 1986) caused a gradual political turn away from nuclear power in some countries (Janzing 2011). The share of nuclear power in Organisation for Economic Co-operation and Development (OECD) research budgets subsequently fell from more than 75% (1974) to less than 25% (2018). However, the research budget share of renewable energy has remained low; it has ranged below 20 percent to this day (IEA 2019). Even with small but focused budgets, power generation from renewable sources has succeeded.^2^ These achievements are not only due to research, but also to manufacturing and politics.

### Breakthrough in the Bundestag (German Parliament)

The decisive impulse for the success of renewables was not of a technical nature, nor was it a question of resource quality. Rather, the determining factor for take-off was of an institutional nature, based on changes to laws on tariffs and new, open market structures.

An important forerunner was the PURPA Act (1978) of US President Jimmy Carter. It forced US power grid operators to purchase electricity from independent producers at fixed prices. From then on, new suppliers could feed their electricity production into the monopolistically organized grids and sell it at a price of *avoided costs* to utilities.

The first German Electricity Feed-In Act (*Stromeinspeisegesetz StrEG*) was passed in 1990 under chancellor Helmut Kohl and was followed by similar regulations in Switzerland (1991) and Denmark (1993).

The Feed-In Act departed from the avoided costs approach of the US PURPA Act. It provided *technology-specific compensations*. These were based on the *production costs* of each technology and not on the prices of conventional bulk power. The tariffs were set at multiple levels as a fixed percentage of average end consumer prices. This, combined with technology programs from universities and pilot programs, opened the door for the industrial breakthrough of wind power in Germany.

In 1998, the Electricity Feed-In Act was put under reform. The new red-green majority in the Bundestag introduced the Renewable Energy Sources Act (*EEG*) in 2000. The EEG further diversified the levels of tariffs paid to producers. Now the discussion circulated around tariffs at the *cost of generation*. The legally guaranteed amounts per kWh were allowed to be higher than the end consumer price including cost of energy, grid charges, and taxes. To prevent excessive consequential costs, there was a proposed 5 percent annual reduction of tariffs, which later happened much faster.

**Fig. 3:**
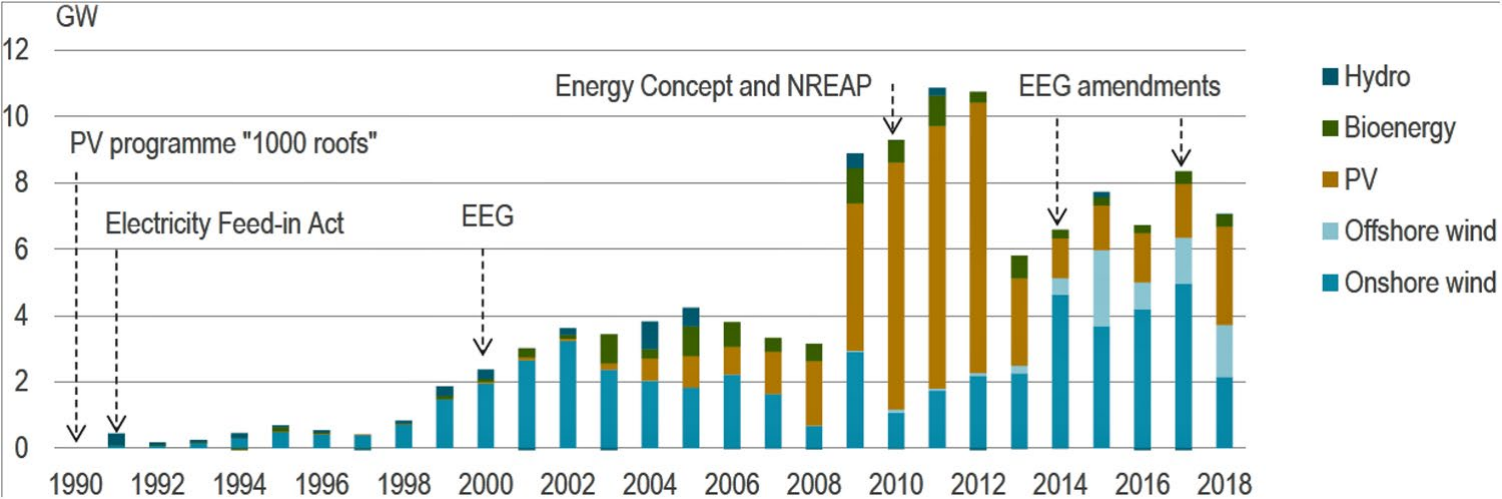
Policy support changes and renewables capacity deployment, 1990–2017 (IEA 2020)

The EEG required utilities to purchase electricity from any renewable energy generator in their area and to concede priority access to the grid. It provided a fixed payment guarantee (cents per kWh delivered) for the full output of an installation over a guaranteed period time, spanning 20 years. The payment amount was re-structured several times according to technology, project size, quality of renewable resource at site, and other variables that affected project economics (Fig. 3).

In 2004, the feed-in tariff for solar power was set at € 0.574/kWh for 20 years for all new installations starting production in that calendar year. A calculated profit on equity capital of 7 percent per year was used as a measure taken from good practice reference installations.

**Fig. 4:**
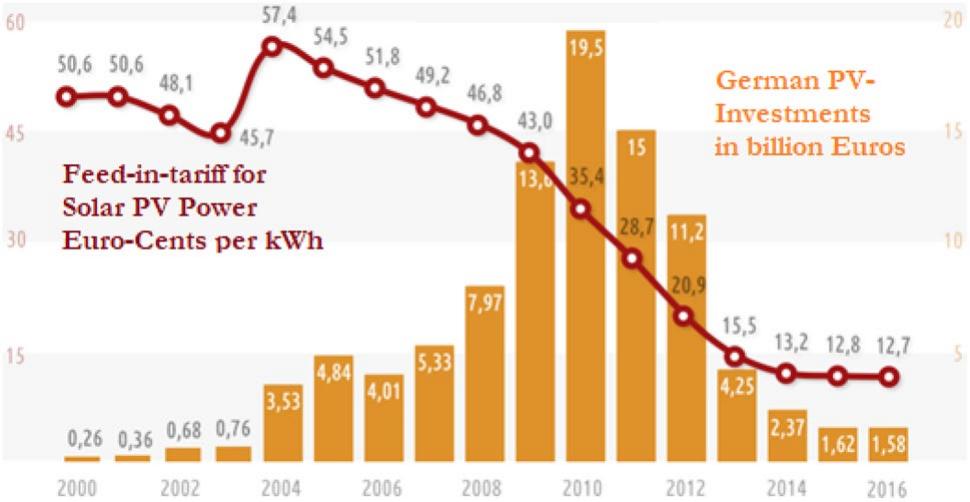
PV systems in Germany: feed-in tariffs and investments 2000–2016 (Stromreport.de, 2020)

Thousands—and later hundreds of thousands—of ordinary citizens began to invest in decentralized solar and wind power plants. A market worth of billions of Euros was created, with several thousand megawatts of new capacity added each year (Fig. 4). This largely happened outside of the old, oligopolistic electricity industry, which continued to invest in coal- and gas-fired power plants.

**Fig. 5:**
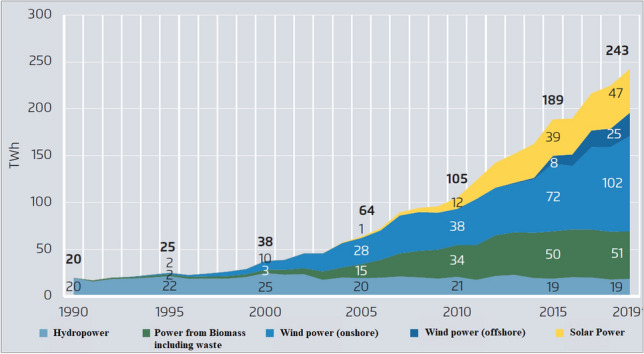
Electricity generation from renewable energies in Germany 1990–2019 (Agora 2020)

Thanks to minimum prices, new technologies including wind, photovoltaic, geothermal, biomass, and (later) offshore wind power could grow at an industrial scale when they were not profitable before. From 1990 to 2019, German power production from renewables increased from 20 to 243 TWh per year (Fig. 5).

### Creation of avoidance industries

The scope for legal remuneration was based on certain primary energies: wind, solar, hydro, bio, geothermal. Within these renewable primary sources, feed-in tariffs operated technology-neutral. No grants were paid for the construction of new power plants. Only real delivery was paid for *power from new fields of technology* that were perceived as avoidance industries.

The price guarantees were open to deliveries from all competing sources within the legally defined boundaries. It made no difference whether solar electricity was generated using solar thermal technology or photovoltaics; in the case of wind energy, both horizontal and vertical turbines were entitled to remuneration, or those with two, three or four rotor blades.

The new renewable technologies differed from nuclear power. Generation was distributed and generation costs were falling. Small and medium-sized companies in Germany, Denmark, Switzerland and elsewhere were involved in manufacturing and in developing and refining components. This bottom-up approach, the technology neutrality of remuneration and the open access to markets provided for a new industry that had few administrative barriers. There were also risks when, for example, years after the introduction of the German Renewable Energy Act (EEG), cheap Chinese solar modules arrived on the German market, outcompeting German market leaders. Within the new supply of technology, investors selected the best offers. Some companies were successful; many pioneers fell by the wayside.

### The concept of dynamic efficiency

The parliamentary initiators of the German Renewable Energy Act (EEG)—first and foremost members of parliament Hermann Scheer (SPD, 1944–2010) and Hans-Josef Fell (The Greens, *1952), together with so many others—recognized from the outset that it would be wrong to take *current costs and prices* of power generation and efficiency technologies as a yardstick for economic efficiency and political aims. Rather, the goal was to increase market share and bring down costs of a large range of environmentally friendly technologies over long periods of time. Cost reduction was, of course, a primary goal, but it was not the only one. Equally important were a more democratic, bottom-up access to and control of energy supply and a wider diversity of actors within a decentralized generation and distribution structure. Policy should no longer protect extractive and polluting industries, rather, it should honor the prevention of further environmental damage.

Hoping for learning curves bringing cost reductions, the term "efficiency" was not considered static and based on pure cost indicators, but instead, dynamic (Fritsch et al. 1993; Krey et al. 2000). Higher economic costs for consumers in the short and medium term were tolerated. The additional costs of feed-in tariffs were passed on as a surcharge on power prices for final consumers. The sharing of this burden, too, later led to fierce discussions, political conflicts and increasing exemptions for the business sector.

It was no surprise that feed-in tariffs, initially up to ten times the actual market price, were harshly criticized by neo-liberal economists in Germany and by advocates of the conventional power industry who made current prices the measure of all things. They attacked the environmental and innovation goals of the former ruling red-green parliamentary alliance, which looked to overcome path-dependency and lock-in effects of the nuclear and fossil fuel industries.

Minimum prices paid by consumers were denounced as a “subsidy,” and the law was framed as a deep "sin," ignoring its aims for long-term ecological benefits, innovation and cost reductions. It was demanded that "money should be used to insulate houses better, which in turn is most cost-effective with polystyrene" (Pfeiffer et al. 2020).

Some economists criticized the “inefficient technology portfolio” covered by feed-in tariffs and argued for a quota scheme instead. Many of them advocated for abandoning feed-in tariffs entirely (Weimann 2013). The law was blamed for trying to serve "far too many unrealistic hopes." By abolishing feed-in tariffs, electricity would become cheaper and CO_2_ emissions would anyway be capped by the European emissions trading system (Sinn 2012).

The location of power generation could be more efficiently organized by a concentration of wind power in Northern EU regions and of solar power in Southern EU member states and by harmonizing renewable energy policy within Europe (Haucap 2012; Frondel et al. 2013; Mundt 2013). Efficiency was a main argument of the European Commission, too. “The historical development of renewable energy systems policies in the EU has shown, above all, that member states consistently resist the Commission’s attempts to implement an EU-wide quota scheme.” A majority of member states were countries with second-best resource quality at best, compared to the sunshine level in Southern Spain or wind resources in Northern Scotland, Sweden, Denmark or Norway. A quota system threatened an immediate and risky shift of electricity infrastructure toward regions far away from load centers in Central Europe. Governments, grid operators and EU institutions were not prepared for this at the time. The support for expensive photovoltaic installations in Germany might have ceased completely with a shift of installations toward Italy or Spain. Both EU directives (2001/77/EC and the substituting directive 2009/28/EC) have been interpreted as failed attempts to introduce EU-wide quota systems (Strunz et al. 2014).

Objectors to feed-in tariffs ignored the fact that under simple quota systems, private actors failed to expand innovative technologies such as offshore wind or PV. A quota scheme would have grown only the cheapest technology which at that time was onshore wind power. Quota systems would have led to a concentration of plants in a few regions, as earlier examples from Great Britain showed. They risked accelerating local externalities and threatened landscape protection, biodiversity (birds) and noise pollution control while accelerating “nimby” behaviors.^3^

Feed-in tariffs were the decisive choice for addressing a high diversity of supply and a decentralized, “home-grown” power system with popular investment participation. In this way, a higher diversity of supply was also guaranteed with regard to summer–winter seasonality and daytime–nighttime load profile difference. The combination of solar, wind, hydro, biomass and new storage technologies had system benefits compared to a pure technology-neutral deployment of least-cost technologies that disregarded security of supply and grid extension failures.

The market prospects of PV and other technologies that were in stages of early development would not have benefited from a quota scheme. Ultimately, “the feed-in tariff-driven, large-scale deployment of photovoltaic installations in Germany during the last decade contributed to driving down module costs” (Strunz 2014).

When, after the nuclear accident in Fukushima, the German (right-wing) majority coalition confirmed the closing of all nuclear power stations by 2022, this aroused opposition. Some critics simply resisted technological change and disguised their aversion against renewable energies in pseudo-economic arguments. Others feared the market backlash of their main facilities. The methods of the nuclear and fossil lobbies were similar to the PR strategies of the tobacco industry (Brandt 2012): Industry-related "think tanks" fed the media supposedly “scientific findings.” These appeared on TV shows and in industry-friendly newspapers that continued to deny the risks of nuclear energy or climate change.

For more than two decades, the energy transition was derided as pointless, unattractive, technologically impossible or unaffordable (Eisenring 2017; Sinn 2012; Frondel et al. 2014; Buck 2018; FT 2014; Butler 2018). Failed pilot plants and pioneer companies were publicly gutted: in Germany, for example, the wind power experiment "Growian" (1983) and in the USA, state support for the solar company Solyndra, which went bankrupt in 2011.

Still, many countries outside the EU, including Switzerland and its small consumers, do not have freedom to choose suppliers or competitive power markets. Thus, it is no surprise that fossil and nuclear lobbies continue to blame the *Energiewende* for allegedly unresolved problems or costs. They hope to continue their harmful operations by looking for government protection or new clients in monopolistic power markets.

## New (old) arguments and real performance

Wind and solar tenders proved to be a cheap—or the cheapest—choice in many power markets, compared with new or existing coal, gas or nuclear generation (BNEF 2020; Wamsted 2020). But controversies over the transformation of energy systems continue to this day on local and global levels. New (old) issues of opposition arose and were promoted by lobbyists. This section deals with these alleged issues and discusses each one:

1. Due to the expansion of generation at new sites, grid congestion would increase. Congestion management costs grew 74% in Britain and 14-fold in Germany (Joos 2018). The allegation was that *grid management costs would become unaffordable*.
2. Depending on weather, solar and wind operate at lower capacity factors compared to coal or nuclear plants. The allegation was that *security of supply could not be guaranteed* except by *permanent reliance* on fossil fuel backup systems.
3. Low figures for levelized cost of energy (LCOE) did not tell the whole story: “To accommodate variable renewable power output while enforcing high standards for security of supply, *costs are incurred in other parts of the system*, mainly for holding and operating reserve and back up plants to manage variability and uncertainty of VRE [variable renewable energy] output” (Joos 2018).
4. Germany was portrayed as a “ghost driver in the nuclear phase-out” because wind and solar power were “generated stochastically.” This *electricity would be "so worthless* that the electricity prices in Germany are sometimes negative" and “the technical equipment needed to collect the energy in the area” would be “very expensive” (Sinn 2012).
5. Critics then denounced the transition as “*socially unjust*” and support for renewable energy to be a prevailing *electricity price driver* that resulted in benefits mainly or exclusively for the "rich," a "cost tsunami" (Frondel et al. 2013; Haucap 2012) and a "ticking sociopolitical time bomb" (Frondel/Summer 2014)
6. Finally, as the European Commission communicated in 2014, a shutdown of nuclear power plants would likely result in a “*higher use of gas and coal*,” compromising EU climate goals (Sopher 2015; Helm 2017).

1. Grid congestions

Grid congestion and curtailment costs are not specific to Germany, nor do they arise from renewable power only. Adjustments of generation portfolios are regularly followed by the adaptation of grid architectures. In Switzerland, for example, during baseload build-out of nuclear power plants, distribution networks had to be enhanced for additional electrical heating systems to get rid of power surpluses at night. This caused costs that were socialized, without lobby complaints, by an increase in uniform tariffs for all small consumers including consumers without such heating appliances.

Transmission System Operators (TSO’s) all over the world manage grid congestion. They arise in emerging economies when power consumption grows at high rates. In OECD member countries where power consumption has been regressing for some time and new technologies have been expanding, grid congestion can be an indicator for complicated planning procedures of provincial or local administrations that are not in line with national climate policy.

Opposition against build-out may be indicative of lack of communication, lack of participation or missed technological innovation such as underground cabling or the introduction of megawatt-sized batteries. It also cannot be excluded that incumbent power plant owners with non-renewable capacities have been quite happy with grid extension delays. This way they have gradually been able to protect their supply regions against cheap wind power deliveries from other regions.

“Curtailment” is power that could be generated but was not dispatched onto the grid in response to grid operator requests. Curtailment rates for wind power in Germany rose to a peak of 4.95 percent in 2015 (Joos 2018). Higher rates—“15–16% for onshore Scottish farms”—are reported from Scotland (Joos 2018), Texas with up to 17% (DOE 2018) or China with 7% curtailment in 2012 and a much higher estimated peak in 2016 (Radowitz 2019; Huenteler et al. 2018).

**Fig. 6:**
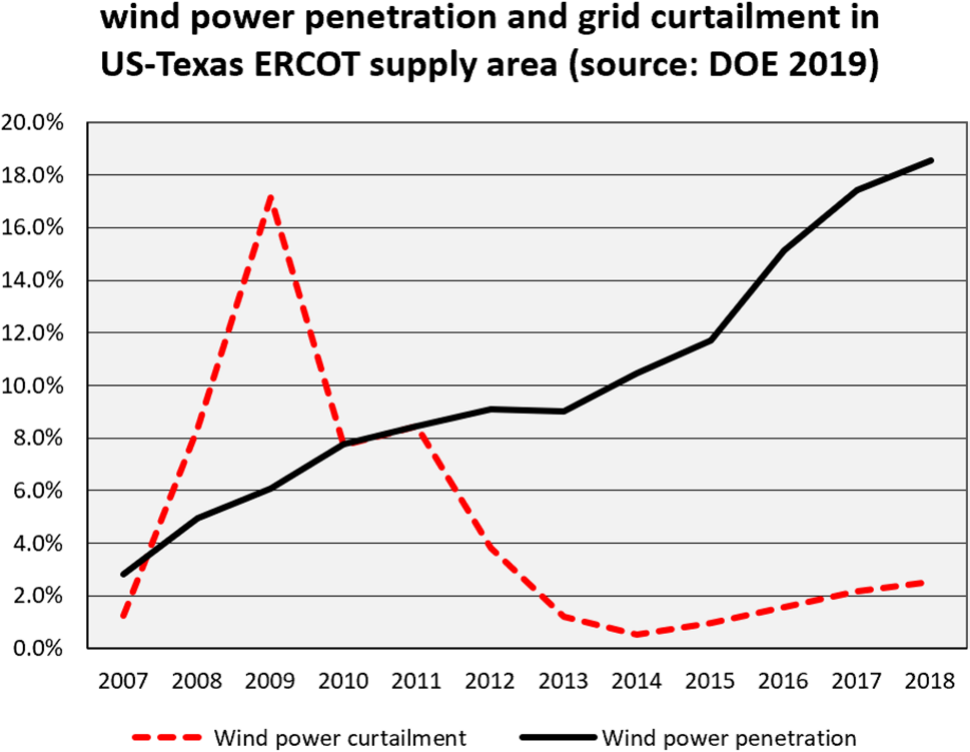
Grid penetration and curtailment of wind power in Texas (ERCOT area) *Source*: US Department of Energy (2018)

Curtailment rates in the USA are well documented over long periods for all transmission system operators. In Texas, extensive wind expansion was never an eco-friendly affair. Rather, it was “business,” and related to a preference for home-grown energy. Wind power curtailments in Texas rose up to more than 17 percent in 2009 with a wind power penetration of only 6 percent (Fig. 6). This problem was resolved by very solid planning for grid extensions and upgrades within a few years (ERCOT 2008). In 2018, the Texas share of wind power supply stood at 18.55 percent of supply and curtailments were reasonably low (2018).

It is important to insist that for economic reasons, it makes no sense to extend grid capacities to 100 percent of wind or solar delivery because production at nominal capacity rating occurs very rarely. German grid regulations allow TSOs to tolerate curtailments of up to three percent of overall output in their network development plans, to avoid disproportionately high grid investments (Joos 2018).

In Germany, just under 2.8 percent of renewable energies were curtailed in 2019; more than 97 percent of renewable generation was delivered to consumers (BnetzA 2020). Grid extensions and grid enhancements were responsible for this reduction of curtailment rates. In 2012, BNetzA approved 2800 km of new lines and 2900 km of additional network enhancements. Lines from the north to the south of Germany were needed to eliminate bottlenecks and to avoid unscheduled “loop flows” that congested the borders with Germany’s neighbors (Sopher 2015). In 2017, the Thüringer Strombrücke transmission line came into service, connecting Saxony-Anhalt and southern Germany. It provided relief and lower re-dispatch costs. (IEA 2020).

By 2020, 7700 km of transmission grid expansion, including four major north–south high-voltage, direct current (HVDC) lines, has been adopted into law. Besides accelerating grid expansion through an updated review process, the strategy aims to enhance existing transmission infrastructure through grid optimization (IEA 2020).

The costs of network and system security measures in 2019 were 1.2 billion euros (2018: 1.4 billion euros). These costs cannot be considered excessive.

(2) Security of supply

Endangered security of supply is a recurring threat communicated in German mass media (Flauger 2019; Wetzel 2019). For instance, they alleged that the rapid boom in fluctuating renewables would confront grid operators with challenges that would only increase with the phase-out of coal-fired and nuclear power generation.

**Fig. 7:**
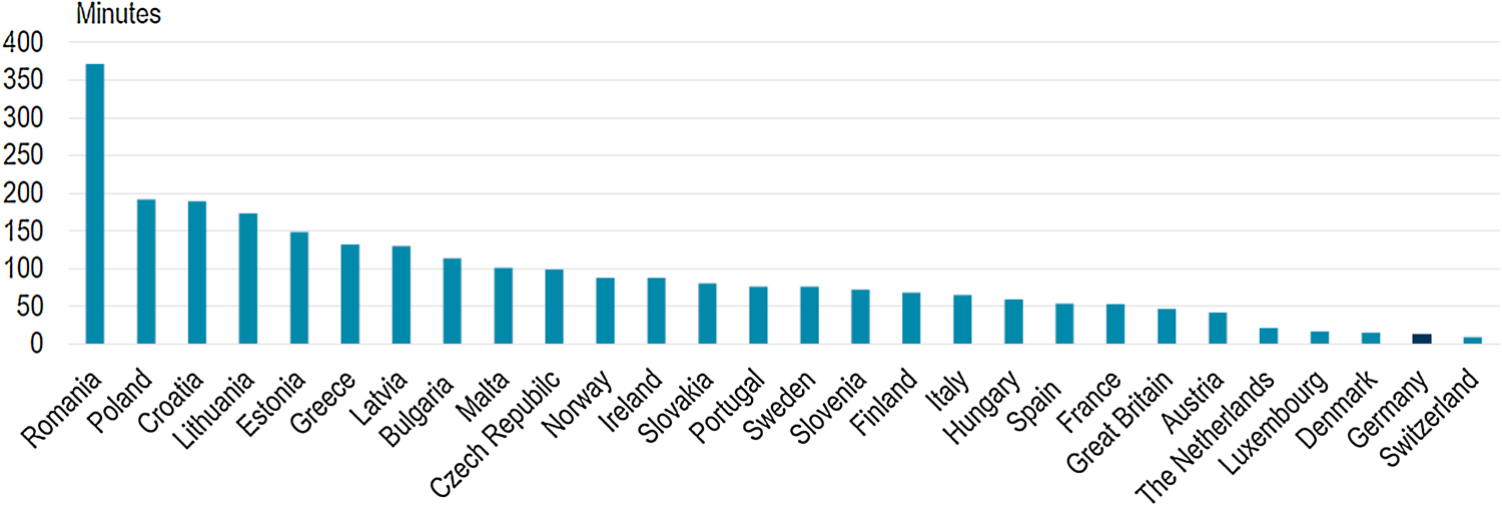
Average annual power supply disruptions in European countries, 2016 (IEA 2020)

So far, however, Germany has one of the highest levels of supply security internationally. The *System Average Interruption Duration Index* (SAIDI) of Germany went from 18.67 min in 2006 to 10.45 min in 2015. German grids have “maintained or even increased their reliability during significant increase of variable renewable energy penetration” (Joos 2018). Interestingly, countries such as Denmark and Spain that have a high share of variable renewable energy output have managed to attain good or very good security of supply in terms of measured supply disruption avoidance (Fig. 7).

(3) Excessive additional costs for balancing?

“Numerous studies exist on integration costs, mostly based on modeling” (Joos 2018). However, in the real world, there is no empirical evidence for projections of high additional costs. Contracted reserve capacities have fallen in the German balancing market (Joos 2018). “Empirically […] the German case seems to prove theory wrong: balancing reserves could be reduced “while VRE capacity increased” (Hirth et al. 2015).

In Switzerland, TSO charges for system service costs have been reduced from CH-Cents 0.77/kWh (2011) to CH-Cents 0.32/kWh in 2018 (ElCom 2019).

It is true that built-in system inertia is falling with more wind and solar farms going online and large coal and nuclear stations retiring. Weather-based power fluctuations may cause steeper ramp-up rates and make frequency changes more rapid.

Technological and organizational solutions have been launched to prevent damage or high costs. Most wind and solar farms meet the technical requirements for frequency support; they are able to deliver reactive power and system restoration services as prescribed by grid codes. “Costs for installing the necessary hardware to provide frequency support are negligible (below 1% of turbine capital costs)…with appropriate frequency controllers wind plant could provide fast frequency response similar to that of conventional plant” (Joos 2018).

The four German TSOs established a Grid Control Cooperation (*Netzregelverbund*) which integrated their four balancing areas into one, with joint reserve sizing and activation.

### New technical options for balancing

Where balancing is a problem, it seems that it is “the design of balancing power markets” that “constitutes an unnecessary entry barrier to this market” (Hirth et al. 2015). Opening balancing markets to smaller providers offers important new flexibility and revenue streams such as short-term storage and demand side response. A larger number of providers increases competition and could improve efficiency (Joos 2018).

The European Agency for the Cooperation of Energy Regulators (ACER) is therefore asking for “non-discriminatory, fair, objective, transparent, market based and economically efficient" procurements. Market reforms should eliminate barriers including shorter lead times and product lengths, smaller minimum bid sizes and allowances for pooling.

More recently, investments in big (megawatt-sized) and small (clustered) batteries are emerging in many countries, with Australia and California first. In some cases, they operate at lower costs compared with traditional natural gas peaking plants, depending on gas prices (Newbery 2018; AEMO 2020; Wamsted 2020; Parkinson 2020). Cost effective in certain power markets, they may be a substitute for grid extension. With battery costs continuing to fall and a steep build-out of battery production capacities, a fundamental change in grid management support systems may be expected before 2030.

The conversion from combustion engines to battery-powered vehicles may deliver another new, large cushion for balancing power. The numbers modeled in the context of a mass transition to electric vehicles can be staggering. Let us take the case of Switzerland, where the power hub is blessed with backup power from hydro storage systems. Swiss hydroelectric plants have a combined capacity of 15.51 GW, 2.56 GW of which is pumped storage (UVEK 2020). For years, this capacity and storage played a crucial role in sweeping the power trade with Italy, France and Germany.

Switzerland counts 4.6 million private passenger cars (Federal Statistical Office 2020). If these were replaced by battery-powered vehicles, a new pool of grid-connected batteries could be made available. Assuming that each car has a configuration similar to Tesla's Model 3 (340 kW output, 75 kWh energy storage), the additional power capacity would amount to 1564 GW, corresponding to 100 times the capacity of all hydroelectric power plants combined or 600 times the capacity of pumped storage. These numbers show that even a marginal use of vehicle-to-grid appliances can substantially contribute to smoothing frequency control and power management, if technical specifications and financial incentives are in place.

As with grid congestion, there are no built-in technical barriers to the accommodation of control power for high volumes of power from renewable energy sources. It is a question of taking responsibility for investments and the adaption of power markets. “In summary,” integrating large amounts of wind and solar energy into power systems is “less dramatic than sometimes believed” (Hirth et al. 2015) and “has not proven to be the major impediment that was once feared” (Joos 2018).

(4) Unaffordable profile costs?

The temporal mismatch between variable renewable energy output and the load results in profile costs. The cost can be regarded as diminishing cost savings from the substitution of thermal plants by renewable energies (Hirth et al. 2015).

Weather-driven output fluctuation requires adjustments to scheduled operations and the utilization of residual backup systems. For incumbent companies with thermal power generation, that means increased ramping, cycling, partial-load operations and reduced utilization hours. These adjustments cause additional costs.

However, reducing thermal generation and reducing externalities are main goals of the Paris Climate Agreement. This change entails short-term costs to prevent long-term damage.

Constraints to storage, plant flexibility and grids make electricity a heterogeneous commodity with varying economic values across time, delivery lead time and location. This results in technological, institutional and managerial challenges associated with grid operation, such as the increased need for flexible resources (e.g., flexible plants, storage, demand response, grid infrastructure) and power quality control, better interregional coordination and sophisticated methods of sizing reserves (Hu et al. 2018).

Each contributing element of power management may have its own merit order. New storages, demand-side flexibility and additional grid infrastructure should be based on technical, resource and cost deliberations. To optimize these activities, each element should ideally be accessed at equal marginal costs.

Some countries in Europe are blessed with rich natural resources such as hydropower storage or large volumes of biomass. The Swiss hydro and pumped hydro capacities cover 180 percent of maximum domestic load demand and are marketed as part of a transnational supply management. Many other countries will have to rely on batteries, power-to-gas systems or interconnections with existing hydro storage such as in Scandinavia.

### Backup for renewables by renewables

Due to wind and solar conditions in Europe, high solar irradiance and high wind speeds have a negative correlation on all time scales from hours to months (Wirth 2020).

An important element for the reduction of profile costs is the use of a diversity of renewable technologies including specifications for more balanced production from specific solar panel orientation, use of bi-facial modules, module tracking and dispersed site locations based on interchanging weather patterns connected over large areas. Using the diversity of weather conditions, different renewable sources can serve as mutual backup to reduce storage demand and dependence on fossil fuel backups.

In this perspective, the nature of renewable energy as a distributed resource turns out to have its own merits. The conclusion from this should be that tenders for new energy procurements should take demand profile into account to a much greater extent than in the past, without leaving the path of technology neutrality. Splitting tenders into different markets and regions using technology-profile/site/orientation for better coverage of seasonal or daily demand profiles should be further investigated.

Additional impacts can be expected from sector coupling where power delivery to electric vehicles, heat pumps or heat stores is structured in a supply-oriented way. All of these new designs may enable wind and solar power generators to increase their market value, while they serve as a cheap source of clean energy for their customers.

Weak carbon prices under the EU ETS scheme before 2018 were highly insufficient for internalizing climate externalities. These low prices also decreased the market value of wind and solar power because the merit order stayed flat, while cheap coal power could be added at any time without considering externalities. Similarly, explicit and implicit subsidies for fossil fuels and price caps in the power market put renewable energy investments at a competitive disadvantage. If these subsidies are not removed, the business case for renewable energy investments remains unsound. (Hu et al. 2018).

(5) Negative prices through incorrect incentives?

Critics of feed-in tariffs concluded that feed-in schemes can “disincentivize variable renewable energy generators to maximize their market value” (Hu et al. 2018). These schemes may inefficiently increase their own policy costs.

While it is correct that initial feed-in tariffs fully shielded wind and solar installations against spot market price signals, weather conditions cannot be changed. To a certain extent, wind or solar power generation will always depend on primary sources of energy, irrespective of siting, locational spreads, diversity of orientation or interconnection capacities. We will therefore have to live with recurring low-price periods. This should be perceived as an opportunity rather than a burden from a consumer perspective.

A growing number of hours or days with negative prices may be an indicator for low market integration of renewables and deficits in sector coupling. Wind and solar facilities can be programmed to curtail their production without ramping costs. It is fossil fuel-based power generation that causes negative prices where ramping costs are too high to curtail production in times of surplus power.

The call for market-based feed-in *premiums* instead of fixed tariffs, launched early on by the EU Commission, was not unheard of (Hu et al. 2018). From 2012, feed-in tariffs were replaced by premium systems in Germany and elsewhere. Obligations for direct marketing of large-scale production were introduced step-by-step, followed by tender systems with floating premiums in 2014.

From an economist’s perspective, the question to ask would be: Which power system’s overall costs, including integration costs and externalities, are lowest?

In 2018–2020, tenders of large-scale wind and solar deployments resulted in contract prices of €0.04 to €0.06/kWh (BnetzA 2020). This was lower than prices for new coal or gas generation. In the EU ETS, a new market stability reserve regulation was introduced in 2017. The prospect of reduced emission permit volumes led to a recovery in CO_2_-prices in the European power market. This also made many of the old coal-fired power plants unprofitable and may have created room for storage investments as a substitute for coal-powered backup.

In Germany, smaller plants (< 750 kW) still get a fixed premium that is somewhat higher than the estimated €0.06/kWh power generation costs of new coal power plants. For these small-sized PV plants, positive externalities may justify the additional cost. Small installations can be integrated into buildings or infrastructures without taking up unused open space. The siting of these installations very close to load can save grid expansion costs. Short distances can reduce transmission-related energy losses and risks. Distributed facilities may advance sector coupling for heating and traffic without charging transmission lines for power. Advancing sector coupling may help stabilize prices caused by variations in weather conditions. The robustness of the system will be improved by smoothing generation and consumption at a very low grid voltage level when main grid congestions affect distribution grids (BnetzA 2019). The positive external effects by a more decentralized power generation architecture are significant for system security, even if they cannot be easily quantified in monetary terms.

For integration of the growing output of German solar PV power at midday, the ramp up of demand in the evening could be perceived as an outlet. Coal and gas-fired power plants would then be replaced by batteries as is done in the USA or Australia where large batteries have started to play a major role in replacing natural gas peak plants as a backup (AEMO 2020; Wamsted 2020; Parkinson 2020). In Germany, costly thermal back-up capacities could then be mothballed over longer time periods, while periods of negative prices could be reduced. In this regard, the speed of price reductions for stationary battery systems and their future availability must be considered.

(6) A “sociopolitical time bomb”?

Initial costs of the German *Energiewende* were substantial. In 2014, the European Commission estimated that “the expansion of renewable energies reaching a share of 63% by 2030 would result in additional costs of EUR 137 billion compared to a fossil-fuel based reference scenario” (Sopher 2015). Estimates by Bloomberg New Energy Finance put the total cost to date of Germany’s clean energy expansion at €106 billion (Nicola 2014a, b). Meanwhile, however, cost reductions have continued to an extent that was not expected. Indeed, a large part of the costs seems to be transitional and non-recurring.

Cost reductions can be expected soon after 2020, and their causes can clearly be identified:

- PV installations with particularly high feed-in tariffs started right after the year 2000; they will lose their entitlements after 20 years of operation. This will start in 2021, and it will generate cumulative cost relief.
- Many of these early installations will continue to operate at lower benefits from the spot market prices; they will contribute to the merit order-effect by displacing more expensive thermal plants and contributing to consumer relief.
- The end of feed-in tariffs and much lower costs for new generation facilities can be observed for all technologies with EEG-premiums. Until 2035, the specific EEG surcharge is expected to fall by 68 percent per kWh of renewable energy output injected (Hein et al. 2020).

It is important to insist that using the EEG surcharge as a yardstick for increased electricity prices is inadequate (Nestle et al. 2012; Gawel et al. 2015). The surcharge is the difference between feed-in tariffs paid and the market revenue from the electricity exchange. The latter is significantly influenced by the delivery of variable renewable energy output which tends to lower electricity prices by the merit order effect, displacing thermal sources of power with higher marginal costs.

A correlation between wind or solar power penetration and the decrease in average spot prices has been shown by a range of empirical studies for Austria, Germany, Italy, Spain or Denmark (Sensfuss 2006; Bode 2006; Sensfuss et al. 2011; Brunekreft 2016; Coester 2018; Hu et al. 2018).

**Fig. 8:**
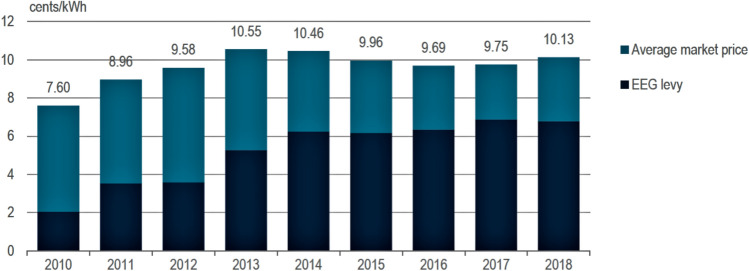
EEG surcharge and average wholesale electricity market price, 2010–18 (IEA 2020)

Thus, the purchase price of a kilowatt-hour of electricity does not increase to the same extent as the EEG surcharge. In fact, power procurement costs for households have been stable since 2013 or have tended to be slightly lower (IEA 2020). It is clearly visible that households have benefited from the energy price reductions on the electricity exchange (Fig. 8) while not being charged additionally after 2013.

The annual EEG surcharge (€25.6 billion in 2018) was disproportionally charged on small consumers. After 2012, more than 2200 German companies benefitted from an exemption (Strunz et al. 2014). Large industries are widely exempt from the EEG surcharge and get exemptions from grid costs, too (IEA 2020). In addition, German electricity prices include a special tax of € 0.0205/kWh (Stromsteuer) introduced in 1999 as an incentive for efficiency that is used for a reduction of payroll contributions for the German state pension scheme.

In these regards, the allegation that power charges are socially lopsided may be justified. Regarding the evolution of overall energy costs however, Gawel et al. (2015) pointed out that oil and gas prices moved higher than electricity prices over the period concerned.

A discussion of reshuffling energy taxes and charges started in Germany when the ruling cabinet launched its 2030 climate action plan (IEA 2020). Major reforms can be expected. It seems fair to give households a relief. This could be done by financing non-recurrent expenditures through the national budget or by using revenues from the EU ETS. Abolishing the tax on power (*Stromsteuer*) is under discussion too. If so, the price hurdle for heat pumps and electric vehicle charging would be lowered.

(7) Additional carbon emissions?

**Fig. 9:**
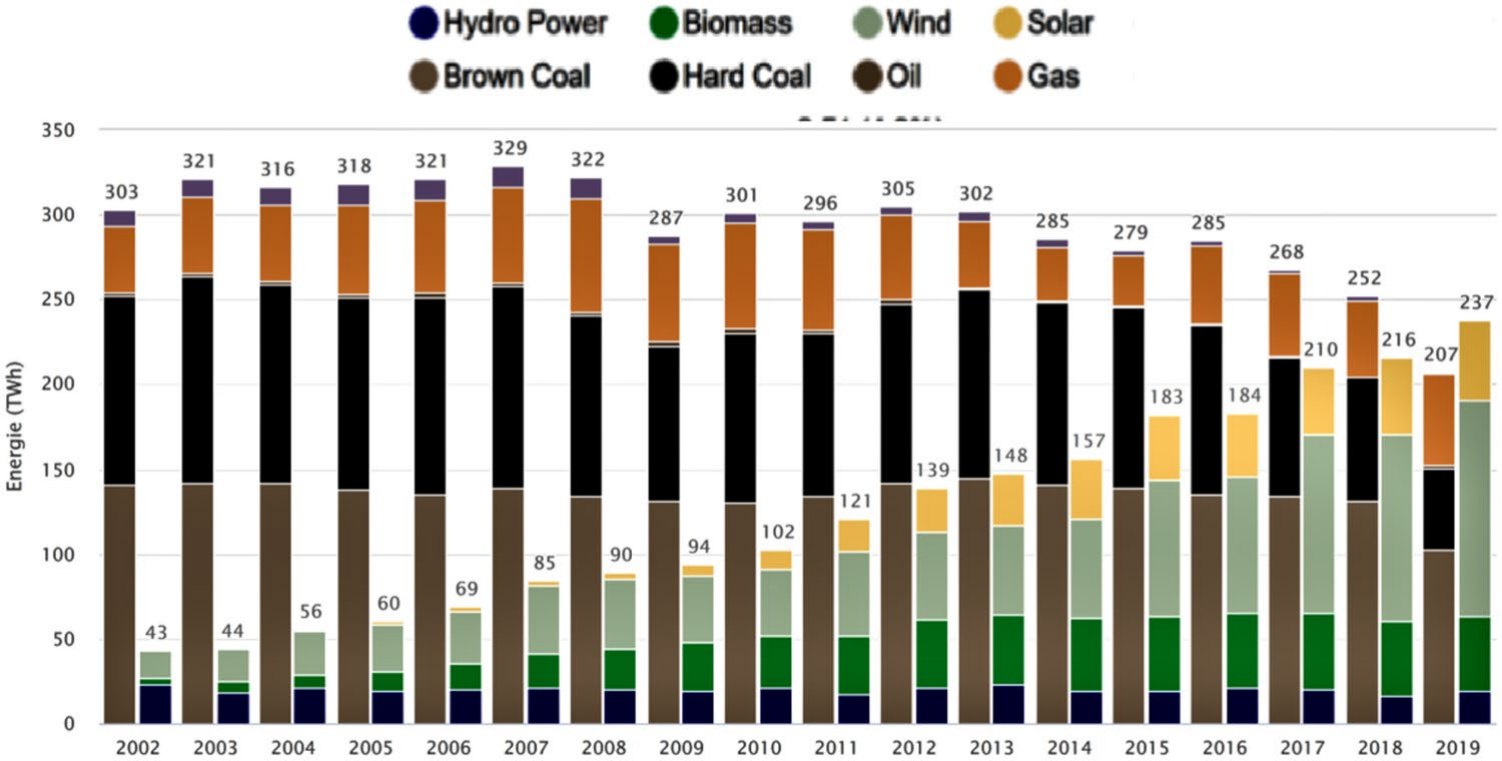
Power generation from fossil and renewable energy in Germany 2002–2019 (ISE 2020)

The discussion in Germany was fueled additionally by the Anglo-Saxon media. They praised the success of coal plant replacements by renewables and natural gas in the USA and in the UK and linked the German nuclear phase-out to an allegedly unstoppable increase in CO_2_ emissions (FT 2014; Buck 2018; Butler 2018). The fact that US methane emissions by natural gas fracturing (“fracking”) increased massively was generously overlooked (Borunda 2020). In 2019, for the first time, power generation from renewable energy exceeded generation from fossil fuels in Germany (Fig. 9) and in the first half of 2020, the share of renewable energies in the German power grid reached over 50 percent. Looking at the period from 2011 to 2020, the accusations made against Germany were not justified. Rather, as far as climate policy was concerned, Germany insisted on a European solution and achieved a successful revision of the rules of the EU ETS in 2017. Meanwhile, the share of renewable energy in the German electricity mix significantly exceeds the shares in the UK and USA; CO_2_ emissions have also decreased (BP 2020).

While carbon emissions from coal plants did indeed remain high for some time, the phasing out of nuclear energy substantially accelerated the expansion of renewable energy investments in Germany after 2011 (Fig. 5). This expansion accelerated further cost reductions and therefore contributed to an accelerated worldwide expansion of clean technologies. Taking all these effects into account, the allegation that the nuclear phase-out led to an increase in carbon emissions does not seem valid.

The phase out of nuclear power is a question of risk perceptions and risk preferences. The Chernobyl and Fukushima accidents revealed that no medical system or liability insurance was prepared for this kind of accident. A majority of the German population continues to be skeptical of purportedly “safe nuclear power.” After Fukushima, 82 percent of Germans supported nuclear phase-out and the increase in renewable energy sources (Strunz et al. 2014). According to Bloomberg New Energy Finance (BNEF), “67 percent think the country isn’t doing enough to move to renewables” (Nicola 2014a). The phase-out of nuclear power seems perfectly in-line with the public majority.

**Fig. 10:**
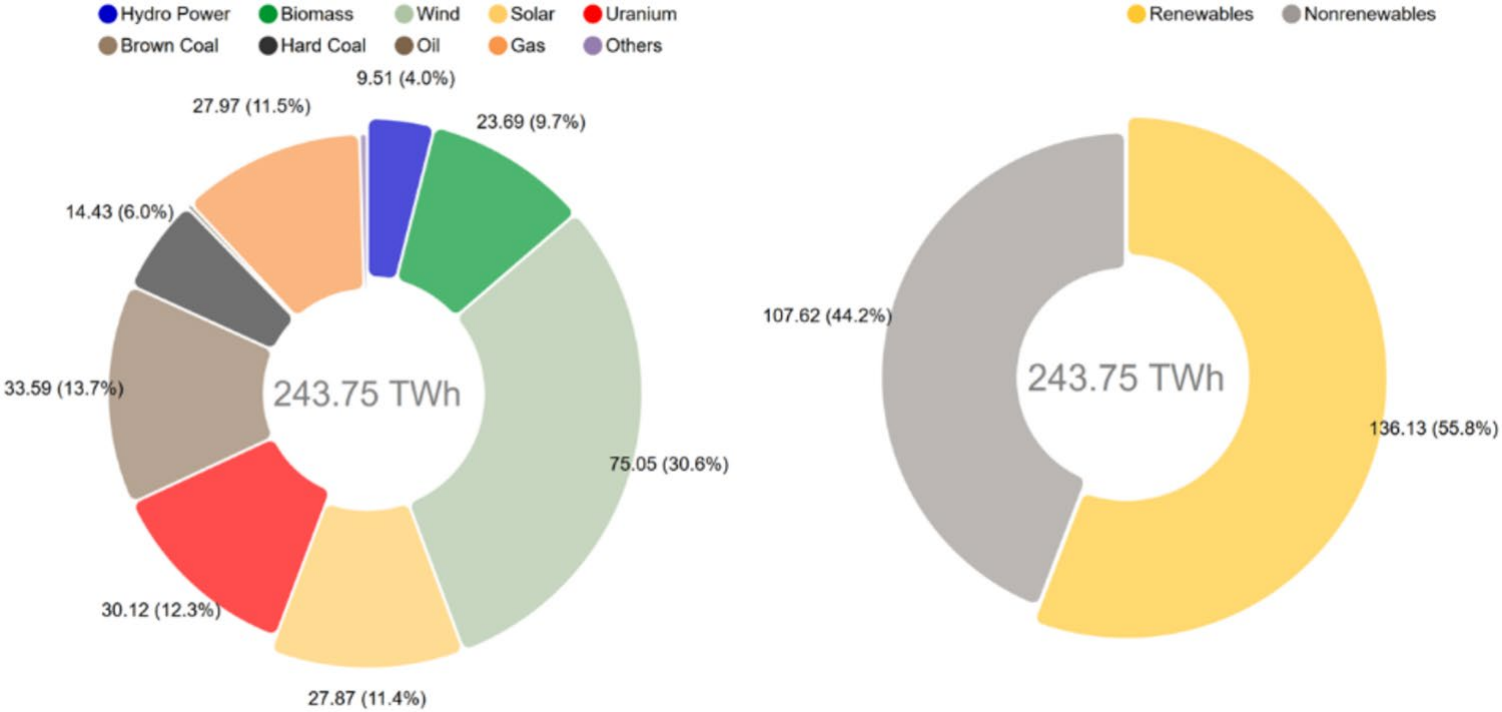
Power generation in Germany, January–June 2020 (Fraunhofer ISE (2020)

Over the first half of 2020, the share of renewable energy in German net power generation was 55.8 percent (Fig. 10), helped by a slight overall reduction in power generation (minus 8 percent) caused by the COVID-19 pandemic (Fraunhofer ISE 2020).

In summary, these developments qualify Germany’s case as a success. While renewable energy sources meanwhile are cost competitive or come in with a least-cost status, the level of security of supply is largely unaffected by the increased share of fluctuating sources. Further expansion may be supported by new technologies such as better batteries at a lower cost. For adjusting the power system to a further rising share of RE and maintaining security of supply, a variety of intelligent solutions will be necessary including adaption of the electricity grid to meet the demands of more decentralized power production, demand-side management, short-term and long-term storage and a higher diversity of tenders where demand profiles can enter as a trigger for remuneration of supply. To make use of these flexibilities, new markets with shorter lead times are necessary. Building of ample storage capacity to reduce intermittency problems, enhanced demand-side management and cross-border interconnections all can be helpful to reduce supply risks and reliance on fossil fuels.

## The relevance of renewables: restructuring and expansion

### Missed opportunities

The falling costs of renewable power increased the acceptance of “clean power” worldwide. Many new markets with no ecological ambitions started to grow exponentially due to low prices. But these market penetration successes did little to change ideological positions and financial bribing (Papyrakis et al. 2004; Funk 2020).

### A history of contentious policy reforms

Political resistance against renewable energy technology has a history. The Californian wind boom (1979–1986) was based on tax credits which expired under US Republican President Ronald Reagan (Rao 2019, 666). The Danish wind boom (1990–2000) was stopped by conservative Prime Minister Anders Fogh Rasmussen who abolished the feed-in tariffs right at the start of his term. The Spanish wind boom (1997–2012) came to a spectacular halt in 2013 when conservative Mariano Rajoy (Partido Popular) started his term as Prime Minister (Pekic 2013). Rajoy then introduced a "sun tax" in 2015 to threaten self-consumption by households and small businesses, which was immediately scrapped when a socialist government took over in 2018 (Binnie et al. 2018). These abrupt policy changes came at a cost, reducing or erasing market positions of home-based renewable energy industries and weakening research programs.

These days, renewables are still controversial, even in Germany. Opponents try to stop the energy transition by appealing site permits. Incumbents and grid operators who themselves have had interests in coal or nuclear power play waiting games with grid extensions and protect their traditional supply regions. Discriminatory fees charged for grid connection or on distributed power for self-consumption are a major problem, though addressed by the EU Directive (EU 2018/2001), the so-called clean energy for all package (EU 2018; Rechsteiner 2016). Ruling governments with ties to the fossil fuel and nuclear industry keep auction volumes low or have started to invent other discriminatory practices (Lee 2020a, b).

The late Hermann Scheer, member of the Bundestag and president of "Eurosolar" visited China repeatedly in the early 2000s and pleaded for an introduction of feed-in tariffs and for the expansion of renewable energies. Recognizing the strategic importance of renewables, Chinese leadership introduced feed-in tariffs in 2011 and provided financial support for many solar companies.

With a history of reluctance toward renewables, the main ruling Christian-Democratic party (CDU-CSU) failed for a long time to transfer the success of renewable power generation to the transport and heating sectors. One of the leading anti-wind-protesters in 2020 was an employee within the German ministry for economics and energy (BMWi), led by conservative energy minister Peter Altmeier (Schaudwet 2019). Altmeier tried to impose prohibitive minimum distance rules for wind energy but was hindered by his coalition partner (SPD).

It was a US entrepreneur, Elon Musk, who, thanks to his visionary power, personal fortune and supported by California's ecological ambitions, led the breakthrough of electromobility and later put the German car industry under pressure to adopt more sustainable technologies. Previously, ambitions of German car manufacturers were focused on falsifying emissions data and developing IT-malware. In negotiations in Brussels, Germany deferred stricter standards for gasoline and diesel engines and thus gambled away its chances to take the lead. At the annual conference of the Federation of German Industries (BDI) in 2007, German Chancellor Angela Merkel declared she would take action "with all the strength I have" against stricter emission standards in the EU, (Döring et al, 2018). These bear raids are still taking a toll today. Germany’s automotive industry must fear missing the boat compared to suppliers from the USA and China, who have gotten their first low-emission vehicle series on the road faster and better.

When a large portion of solar panel production began to be allocated to China, the German government waited at the sidelines. China's strength in renewable energy was not the fault of the inventors of the EEG. Rather, it was fault of the German media and parts of the government that denounced the energy transition as fundamentally wrong and silently accepted or even advocated the shrinking domestic solar industry.

### The long-term relevance of the EEG

Reducing the costs of electricity production on a small, medium and large scale, thanks to renewable energy output, will lead to financial relief and productivity gains worldwide, while at the same time improving the trade balances of many importing countries. Low-cost electricity from locally available renewable sources, with short construction times, will provide an effective cost cap for the price development of natural gas, coal and oil. Renewable energies have properties that will enable them to displace other energy sources in the coming decades. The progress made in terms of costs and storage, including the reduced ecological footprint, contributes to the advancement of a recycling economy with a lowered consumption of raw materials. Clean energy can improve the eco-balance of non-energy material recycling activities too. The usual downcycling may be turned into an upcycling of used feedstocks based on clean power.

There are many characteristics of renewable energies that will contribute to the fundamental restructuring of power and energy markets worldwide:

1. Sun and wind are free goods available almost everywhere. This is also true even considering that availability can be temporary and that some renewables may be harvested more effectively at certain sites than at others. Solar and wind power do not have to be traded internationally. They can be harvested close to load centers, i.e., close to demand, either centralized by companies or decentralized by local players. Unlike fossil and nuclear power generation, renewable energies are not based on scarce extractive minerals; they are available in every country and region. Even a densely populated country like Switzerland could cover its electricity consumption solely from solar modules on roofs, façades and infrastructures (BFE 2019a).
2. Renewable energies deliver their outputs depending on the weather. Therefore, it is necessary to manage shortages and surpluses. This alleged disadvantage and the zero-marginal-cost of output are important drivers for regional networking of additional grid capacities and for access to existing or new storage facilities such as pumped hydro, batteries, hydrogen and sector coupling such as heat storage or electric vehicles. Storage has recorded the steepest cost reduction (Fig. 1), and a tenfold growth of demand is expected for key battery systems from 2020 to 2030 (BloombergNEF 2020).
3. The weather-dependent performance of renewable energies in combination with the opening up of power markets fundamentally changes the behavior of market participants. Power traders and large consumers try to benefit from low or even negative prices by starting to adapt consumption to energy supply cycles. Significant parts of power and energy markets are moving from consumption-oriented generation to generation-oriented consumption. To lower energy costs, many devices and systems such as electric vehicles, heat pumps and heavy industries will be able to program their power purchases accordingly by introducing smart grid devices.
4. Short-term intraday markets are replacing long-term supply contracts. Intraday schedule changes have increased from 0.1 million in 2008 (a volume of 20.5 TWh) to 2.8 million (134.9 TWh) in 2015 for flexible sourcing and adaption of power streams (Joos 2018).
5. In addition to power trading, a new inventory of versatile storage facilities that includes different time ranges and power volumes is being introduced worldwide. Batteries and hydrogen storage systems are important elements for market integration and for maintaining security of supply of renewable power generation while enabling an increase in market share. Moreover, new storage facilities are able to deliver control power and voltage control. Step by step, they seem to conquer all positions previously occupied by fossil power plants.
6. Spot prices on electricity exchanges normally reflect the marginal costs of the most expensive power plant on the grid. They do not compensate for the full costs of power generation, including capital costs and depreciation. Indicative planning including private power purchase agreements and regional tenders are thus required for a sufficient installation of new power plants, including backup.
7. The marginal costs for solar, wind and hydro power are zero. Therefore, renewable power generators displace more expensive power plants whenever the sun shines or the wind blows (merit order effect). Lower average utilization of generation capacity puts additional financial pressure on conventional power plants.
8. Reverse auctions for new power delivery have been established as a new standard global practice in a high number of supply regions (REN 21 2020). The cheapest suppliers of power prevail and will receive fixed remuneration for 15 to 20 years. In many parts of the world, tenders for power provision from renewable energy include storage and backup power as well. Batteries serve short-term, high-frequency storage with day-to-night arbitrage, while hydrogen is starting to be used as a substitute for natural gas, serving longer-term, low-frequency storage or industry demand (Lee 2020a, b). In the west of the USA and in the Middle East, combined renewable-and-storage schemes are reported as a competitive solution today, “undercutting” the price of fossil power generation (Wamsted 2020; Bellini 2019).
9. The costs of backup provisions and system services are further reduced by indicative grid extension planning, as practiced in the EU and in China, linking former rather isolated supply areas with existing trade platforms or storage systems over large areas.
10. Larger market areas, including many small states or nations, can reduce system control and reserve provision costs. Large networks should be considered "ecosystems" of their own. Within such ecosystems of power, market actors act interdependently. For instance, when building new capacities, the decisions of a single state or nation will influence the supply and demand conditions of neighboring linked states or countries. If, for example, new wind farms are built in the Dutch North Sea, this will affect electricity supply and prices in neighboring countries such as Belgium, Germany and France, especially in the winter months when wind power generation is high.
11. Renewable energies are modularly scalable, in contrast to coal-fired, oil or natural gas plants. Solar modules can be sold to a very large number of players. A roof, a balcony or a façade is sufficient to cover parts of demand (Scholten et al. 2020). Self-consumption is interesting because transport costs and taxes can be avoided. These often account for more than 50 percent of power costs.
12. Decentralized storage facilities for power and heat can increase the share of self-consumption. With battery and heat storage systems, favorable opportunities can arise too, through the acquisition of cheap electricity from the spot market at times when consumption is low (at night or on weekends) or when the wind is blowing and the sun is shining substantially or synchronously.
13. New battery technology combined with power electronics can deliver very fast and reliable responses required for ancillary services (Düsel 2020). It offers black-start functionality in case of grid collapse, which is important for countries with weak grid systems as well as for highly industrialized nations where grid extensions are lagging. Batteries can easily be used for steep ramping up and down of load cycles. They alleviate the integration of fluctuating renewable energies by controlling frequency and voltage in the system. Legal frameworks should therefore prepare for central and decentralized storage systems to have access to TSO procurements of ancillary services. If organized in a competitive, non-discriminatory way, decentralized storage facilities may deliver control power and reactive power at the same level of quality as central storage facilities.

### The meaning of electrification of energy systems

Of all renewable energy sources, electricity generation from wind and solar is now the cheapest. Low prices combined with innovations in storage will contribute to an extended electrification of traffic, buildings, manufacturing industry and the extractive industry itself. This has far-reaching consequences:

1. *The electricity grid will become the central platform for energy trading* The demand for fossil fuels and the international trade of fossil energies is expected to drop (Bond et al. 2020).
2. *Local and regional cooperation will be the preferred option* While electric *energy* cost has been on a downward path, the pro rata costs for *power transports* including grid costs, energy losses and system security costs have not been reduced to a comparable extent. In a *ceteris paribus* world, this could mean that for microgrids or for networks on a local level new markets for renewable energy output without grid fees will grow with a promise for cost benefits. Moreover, there might also be political and technical risk perception in handing over strategic amounts of grid-bound power supply to people and regions located at large distances from load centers. To keep these costs and risks low, governments regularly seem to prefer local or regional power networks, even at sites with modest or medium productivity, compared with imports over very large distances, as discussed by the Desertec foundation (Scholten et al. 2016; Desertec 2009).
3. *Electricity trading is different from trading fossil fuels* Although there are storage methods (natural hydro storage, pumped-hydro storage, batteries, supercapacitors, power-to-gas, CAES, etc.), their efficiencies vary, and so do their ranges in time and volume. Furthermore, grid restrictions must be respected. Access to storage facilities with large energy volumes and high capacities—such as Scandinavian or Alpine water storage—will be geographically limited.
4. *Cooperation and interoperability are key for security* Horizontal cooperation between supply areas takes on a completely new meaning due to the fluctuation in energy production caused by weather conditions. Access to new, decentralized storage including electric cars and heat storage systems that reduce power demand over critical periods will complement central power storage facilities. To realize these security benefits, legal experts and system managers must ensure that these additional flexibilities are rewarded proportional to their system service relevance.

## Swiss policy attempts promoting avoidance

The environmental policy history of Switzerland has no comparable success story to the German “Energiewende.” But there are some remarkable, smaller achievements for the sustainable management of natural capital and for promoting avoidance technologies, which in some respects worked similarly to the EEG surcharge.

Switzerland is a small, open economy. The country, with just 8.5 million inhabitants, is too small to offer large markets for new technologies. Companies are positioned as suppliers of neighboring EU and other countries. The export quota of goods and services is at > 65 percent (2019). Swiss companies are experienced in environmental technologies. In the renewable energy business, Swiss companies include ABB, Meyer Burger, Huber + Suhner and Gurit.

Too often, however, new technologies are not applied in the real world. "Industry policy" is a taboo, given a strong aversion to the direct financial support of companies. There is a consensus that "conditions like those in agriculture,” where costs are covered largely by the federal budget, should be avoided.

Greenhouse gas emissions of Switzerland are too high. According to the National Inventory Report (NIR), they have fallen by 14 percent since 1990 to 5.4 tons of CO_2_ equivalents per capita. This number does not include emissions from ships or air traffic. The latter has risen by 84 percent since 1990 (NIR 2020). Kerosene is not subject to taxes charged on other fossil fuels; neither VAT nor the CO_2_-levy have to be paid on it. A plane ticket levy of CHF 30 to 120 has recently gained a parliamentary majority and will be the subject of referendum.

Switzerland is a country poor in raw materials. Large amounts of energy, metals, minerals, and feed are imported. The environmental impacts take place abroad, mainly at the expense of the climate, biodiversity, and water availability. The ecological footprint (including imported goods and services) is larger than the average of European Union member states. In 2015 it was 14 t CO_2_ equivalents per capita. Only about one third of the overall CO_2_ footprint of Swiss residents is recorded in the national inventory report (NIR 2020; Bafu 2020c). Having a "planetary benign" status would mean a maximum emission of 0.6 t per capita (Bafu 2018).

### The role of direct democracy

The polluter pays principle (PPP) is an important goal of Swiss environmental legislation. In Switzerland 50,000 signatures are needed to demand a popular vote (referendum) on a new or revised law of Parliament. In general, it can be challenging to win majorities for new levies or incentives according to the PPP. Even small PPP-charges such as waste disposal fees or CO_2_ levies that are reimbursed by a “carbon dividend” may fail by referendum when not properly explained or based on multi-party agreements. Therefore, it is difficult to enforce the polluter-pays principle in Switzerland.

The origin and amount of donations for campaigns, parties, press organizations, and members of parliament do not have to be disclosed. This has repeatedly led to criticism regarding Switzerland’s failure to prevent corruption (GRECO 2019). Direct democracy makes it easy for financially powerful lobbies such as the automotive industry, aviation, or electricity companies to influence public referendums. However, environmental organizations have also built some power and use direct democracy instruments to launch new environmental policies through so called *people’s initiatives* (100,000 signatures needed).

Swiss environmental policies make use of a variety of different instruments, including promoting and financing innovations. It is easier to pass PPP-measures when cost-effective substitutes are ready for market. Substantial public support for research and development is available for the promotion of innovation and substitutes. "Innosuisse" Agency allots CHF 250 million (US$ 270 million) annually for mixed support of new technologies from private companies. The Swiss Federal Office for the Environment (SFOE) provides a maximum of CHF 4 million to support new technologies to reduce pollution. A technology fund for CO_2_-reductions issues credit guarantees (max. CHF 25 million/year).

### The birth of Swiss environmental policy: The Forest Protection Act

In Switzerland, it took decades for forest protection to be enforced. Action was only taken when it was nearly too late.

125 years ago, catastrophes forced our ancestors to rigorously protect the forest (…) today's forest policy understands the forest as a broad public service (…) Not thoughtless use should be in the foreground, but the benefit for all of us. (Leuenberger 2001).

In the early nineteenth century, municipalities, cantons, and private individuals were owners of forests. "The experts were unanimous, and their message was clear but uncomfortable. The onset of industrialization fueled the demand for wood. Entire forests were sold and then cleared […] In addition, new land was needed for agriculture." (Küchli and Baumgartner 2001) The depletion of forests had many negative implications. It led to soil erosion, loss of land, and in 1834 and 1839 severe floods occurred.

However, numerous decades passed until real action was taken. It was not until 1868 when more than 50 people were victims to flooding that "change of opinion in forest policy" occurred. It was the urban, "economically more developed center who campaigned for the protection of mountain forests. This was facilitated by the fact that freight trains were able to import coal. The forestry law was imposed on mountain cantons against their will. It had a reputation of "disciplining mountain regions, which have shown themselves to be unreasonable and unwilling to accept forest protection" (Küchli and Baumgartner 2001).

"Ultimately, the transition to new, fossil energy sources made the transformation possible, because it removed the high pressure of depletion from forests, that was felt in the eighteenth century. The struggle for scarce resources was alleviated, and this probably increased acceptance of new regulations”. (Sieferle 1998). Agriculture also benefited from coal as a new source of energy, while fertilizers were used to increase productivity, and the pressure on forests decreased. Industrialization shifted the creation of revenue to cities and created new niches for near-natural forests that then fulfilled new functions: near-natural silviculture, forest reserves, natural forest reserves for biodiversity.

Success was possible when the problem moved to top of the political agenda of the young federal state. Conservation had to be negotiated and new institutions ready for sanctions had to be secured; this was achieved by the federal state using its monopoly on violence and on property rights, sometimes against the will of provincial governments or people. Forest rangers supervised the reforestation. Anyone who felled trees needed a permit, and this was only granted if the forest as such was not endangered.

Meanwhile, climate change poses new challenges. And there is no law of nature according to which we always have enough time to learn from damage. "The problem of increasing weakness of mountain forests due to air pollutants, over-fertilized soils, new pests and the rising number of extreme meteorological events in Alpine regions observed since the mid-1980s is likely to intensify. Frequent storms as well as landslides and massive avalanches have caused severe damage.” (Greminger and Jordi 2001).

### Economic instruments in environmental policy

Market economies create new products every day. Competition ensures that market participants have a level of efficiency often imposed by transnational companies dominating the sector. However, competition only works for real costs such as wages, materials or capital. Externalities, so called “social costs” at the expense of third parties, are neither charged nor compensated by market forces. They do not appear in income statements or balance sheets.

Legal measures to make externalities "visible" can improve the overall efficiency of markets. They can incentivize new business models and reduce environmental degradation and loss of natural capital such as clean air, biodiversity or climate stability. By managing natural capital and by including environmental costs, processes, products and services are delivered in a more efficient way. Government action through regulation, rationing of emission allowances (emissions trading) or taxing of externalities may open new markets for innovative products, processes and innovative finance instruments.

### Economic instruments in Swiss legal code

Switzerland has succeeded in introducing a few economic instruments over time. Levies, emission trading and feebates are perceived preferred solutions. Unlike standards, they give incentives for innovation, the adaption of consumption with better efficiency, substitution of hazardous products or investments in new products. However, where emission control and product security are established and need to be ensured with minimum transaction costs, emission standards are indispensable.

A pure incentive mechanism exists as a levy for high sulfur heating oil (1997) and for volatile organic compounds (VOCs, 1998). The levy creates an incentive to reduce VOC-containing products. Receipts are distributed evenly to the population by reducing health insurance premiums, together with the revenue from the CO_2_ levy.

There are not many pure incentive schemes to reduce emissions. The prevailing economic instruments are mixed systems. Levies are combined with earmarking and partial reimbursement such as the “carbon dividend.” “Carbon dividend” is a nickname for the redistributed revenue from the CO_2_ levy which is redistributed uniformly to all residents. Each person receives the same amount, regardless of his or her consumption. The distribution of the levy revenues is carried out by health insurance companies who have the most current address directory of Swiss residents since basic insurance is compulsory for all. The system incurs low enforcement costs. In 2021, the redistributed CO2 levy (“carbon dividend”) combined with redistributed tax on VOC is CHF 87.—per year and per person. The amount is settled against the health insurance premium. The goal is to maintain social balance for people of low income. The earmarked part is spent on avoidance activities and investments, often reducing the tax base and often benefiting those with low incomes, such as refurbishments of old, insufficient multi-dwelling buildings.

There exist many charges that are 100% earmarked, namely, to cover recycling costs or costs of waste disposal. Examples for mixed systems are the performance-based heavy goods vehicle levy (HGVL) for freight transport (2001), feed-in tariffs (FiT) for power generation from renewable energy (2008) and the CO_2_-levy on fossil combustibles including a carbon dividend (2008).

### Levies for recycling and waste disposal

"There is hardly any other country in the world who produces that much municipal waste in relation to its resident population" (Bafu 2020d). This is not surprising given the high household incomes in Switzerland. The recycling rate is at 53 percent. Incineration is mandatory for residual waste, mostly processed by waste-to-energy plants.

From 1997, the Environmental Protection Act gave the government the legal power to ask companies to guarantee recycling, waste disposal treatment and prevention. The law prioritized private sector responsibility—producers, importers and vendors—for avoidance and recycling activities. Recycling is labeled a "voluntary measure"; contrary to the semantics, these activities are not "voluntary." But the business sector can decide how to fulfill legal standards. Where recycling or waste management is deficient, the government can decide on sector- or product- based fees.

Many business sectors ensure the financing of recycling through a common, self-organized effort such as with the recycling of paper, electrical and electronic equipment, beverage cans or cars. The cost is usually a single-digit percentage figure of the total cost per unit. In the case of battery recycling and glass beverage packages, no self-organized agreements were achieved by the business sector. Here a disposal fee on products sold was made mandatory by the government. It is charged as part of the product price.

### Performance-based Heavy Goods Vehicle Levy (HGVL)

In Switzerland it proved to be difficult to reduce the emissions of the traffic sector. The number of heavy-duty off-road vehicles (Sport Utility Vehicles) and heavy motorbikes has grown at a record-breaking pace—most of all in high-income suburbs where terrain is flat. CO_2_-emissions from road traffic have not been reduced as prescribed by law. Nevertheless, transport policy is on the move. The behavior of many consumers has gradually changed, especially in urban and semi-urban areas, due to public transport, parking management, promotion of bicycle traffic and car-free inner cities. Technological innovations such as e-bikes and electric vehicles may bring substantial CO_2_ reductions in the foreseeable future. Financial incentives play a major initial role as has been shown in the heavy goods transport sector.

A performance-based Heavy Goods Vehicle Levy (HGVL) was introduced in 2001 in Switzerland. It is a combined levy charged on heavy transports within Switzerland. Vehicles with a nominal weight of more than 3.5 tons must pay the levy according to specific air emissions, kilometers travelled and nominal vehicle weight. A large part of the revenue is used for investments and support of border-to-border rail systems. The extension and refurbishment of rail infrastructures was a primary objective and delivered side benefits for all train passengers as well as for car drivers whose perceived roads to be less congested.

The policy for a modal shift in transport was strongly confirmed by majorities from a half dozen referenda: a new rail-rail link through the Alps (1992), protection of the Alps (1994), introduction of a performance-based heavy goods vehicle levy (1998), financing major railway projects (1998), further expansion of railway infrastructure (2014).

**Fig. 11:**
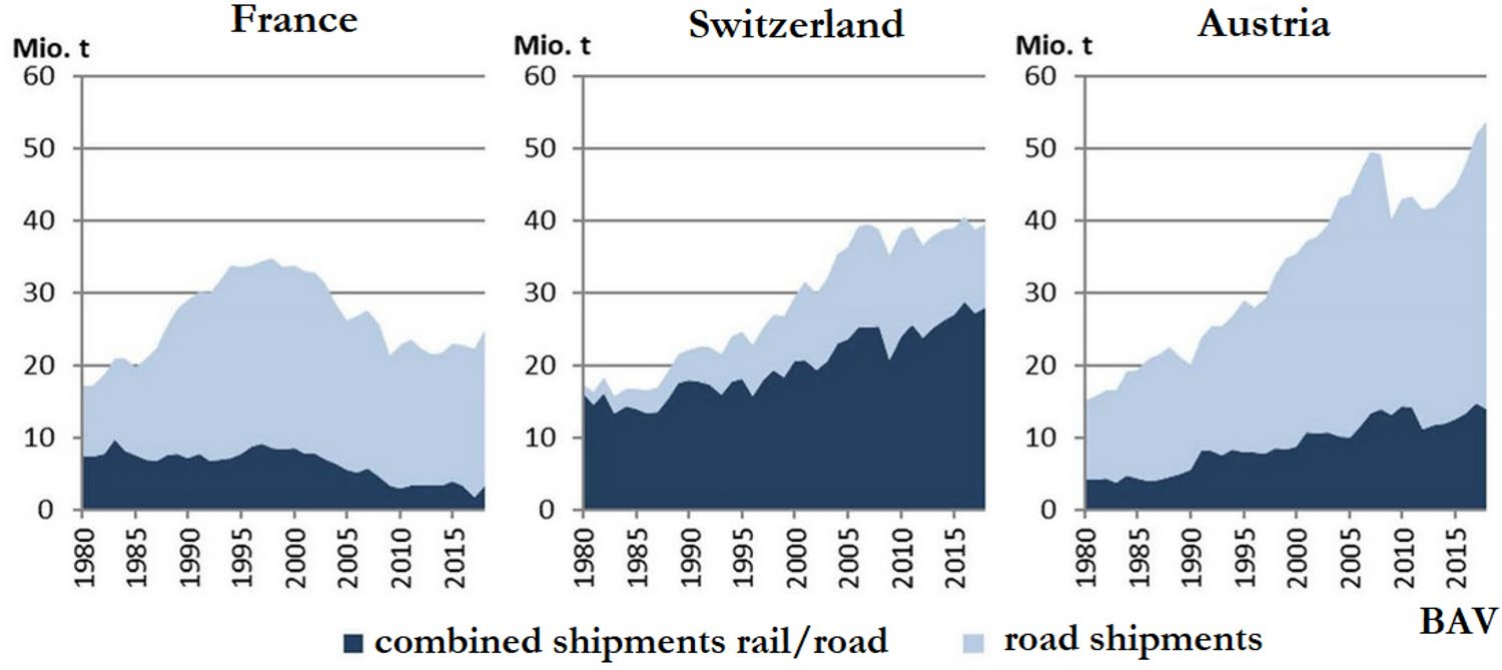
Alpine freight transit traffic: shares of rail and road (FOT 2020)

The number of transits by trucks fell from 1.4 million (2001) to 0.94 million (2018). The train system improved and increased market share after modernizing (Fig. 11). New rail tunnels (Lötschberg, Gotthard, Ceneri) for higher speeds on the Rotterdam-Genoa corridor attracted more clients. The reduction target of a maximum of 0.65 million transits per year may be achieved in some time.

### Feed-in tariffs for electricity from renewable energies

Switzerland was a pioneer in solar energy. In 1991, the city of Burgdorf (Canton of Bern) was the first to introduce a cost-covering feed-in tariff for photovoltaic power generation. Thanks to research, solar companies such as Solarmax (Biel, until 2015) or Meyer Burger (Thun) were established in Switzerland as spin-offs, which earned an international reputation for innovations such as PERC solar cells that are used globally.

**Fig. 12:**
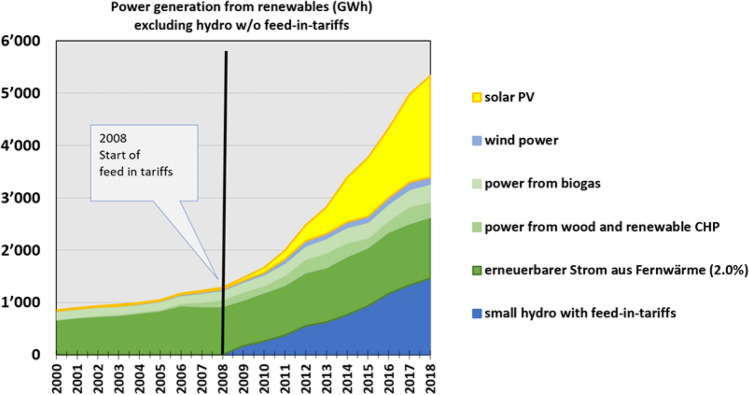
Swiss power generation from renewables (excluding large hydropower, BFE 2019a, b)

Renewable energies suffered a political blockade for decades but despite this some growth was achieved beyond of large hydro power with its traditional high market share of 55–60 percent (Fig. 12). The nuclear lobby feared that the success of solar and wind energy would undermine the public acceptance of nuclear risks. This actually came true in 2017 when a new Swiss energy law prohibiting the construction of new nuclear power stations was adopted in a referendum by a strong majority (58% in favor).

The Paris Agreement on Climate was ratified the same year.

### Combined CO_2_-levy

A pragmatic policy model is the CO_2_-levy. An introduction was first proposed in 1990. It took 18 years until the charge was levied and even then with homeopathic doses of just CHF 12.—per metric ton of CO_2_ (2008). This corresponded to CHF 0.03/L of heating oil and CHF 0.025/m^3^ of natural gas. Over ten years the levy was subsequently increased eightfold to CHF 96/t CO_2_.

In general, much attention has been paid to ensure that levies do not disadvantage export industries. Production facilities with greenhouse gas emissions can be exempted from tax by special arrangement. Exempted companies must in turn deliver an emission reduction commitment or become part of the emissions trading system (ETS). A cooperation between Swiss and European ETS was achieved in 2017. Companies in Switzerland can buy emissions rights of counterparties in the EU. An electronic link enables the transfer of emission allowances between the two systems. The ETS now includes aviation and thermal power plants in line with EU regulations but still, emissions rights may be given for free (Bundesrat 2019).

The CO_2_ levy today is levied on 51% of CO2 emissions from only fossil combustibles (no transport fuels). Around 33% of emissions is regulated in the ETS and 16% comes from companies with target agreements (Bafu 2017). Where the levy has been adopted, two-thirds of revenues are reimbursed to the population by a deduction from health insurance fees (“carbon dividend”). CO_2_-levies paid by industry are reimbursed through deductions from payroll-taxes. One-third of revenue is used for a renovation-of-buildings program.

**Fig. 13:**
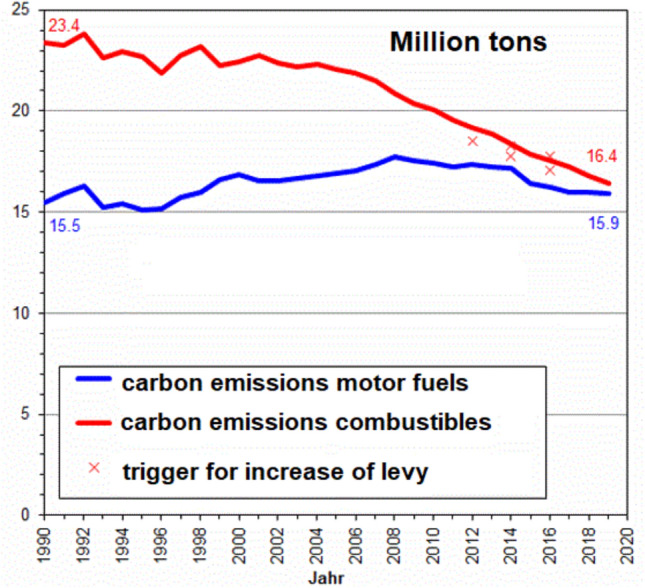
CO_2_ emissions from combustibles and fuels in Switzerland (SFOE 2020b)

Domestic CO_2_ emissions from fossil combustibles in the heating sector have fallen by 29.1 percent (2018) since 1990. CO_2_ emissions from motor fuels have risen by 2.9 percent (aviation is much more, but not included, Fig. 13). Regulatory deficits persist:

- The CO_2_ levy is charged on combustibles only (heating oil and natural gas for heating). Obligations to “compensate” emissions from traffic are not transparent and may be futile.
- No CO_2_ levy exists for fossil mineral inputs of materials such as plastics or cement.
- Companies exempted from CO_2_ levies agree on "voluntary" CO_2_ reductions; these are not yet in line with the Paris Agreement or with the governmental “net zero” goal by 2050.
- Only domestically released CO_2_-emissions from fossil energies are charged. The ecological footprint caused by exploration and extraction activities or the transport of oil, coal and gas (including methane) are not charged. The same goes for emissions from imported goods or services.

### High acceptance for combined incentives

The polluter-pays principle was most well-accepted in recycling, wastewater treatment and waste disposal management. However, some of these charges were often introduced only after treatment facilities and incineration plants had been completed, financed out of general tax revenues.

After volume-oriented levies were introduced, buying behavior changed and waste avoidance activities were observed. This resulted in a reduction of waste quantities and gave rise to political controversy and allegations of bad planning or mismanagement ("pork cycles"). However, there is little point in looking for culprits because those responsible acted within the given frameworks. The mismatch was the price paid to achieve political majorities. Without initial budget financing, the realization of recycling activities or waste treatment installations could have failed in many cases due to resistance (referenda) from populist parties and political entities with tight budgets. These lessons might also prevail in the future.

Earmarked and mixed levies were introduced successfully where avoidance activities were available, could be credibly explained and could contribute visibly to preventing ecological damages: carbon emissions reduction and air pollution control, noise reduction, energy efficiency programs, feed-in tariffs for increasing renewable energy shares and more. The market share of organic farming increased to more than 10 percent mainly based on proper labeling and consumers asking for healthier products; new production lines then started to influence farmer support of policies. The shift of freight transportation from roads to railways reduced noise, pollution, highway congestion and had side-benefits for passenger rail transport. But still, strong infringements on rules persist in climate, drinking water protection, clean air, noise and biodiversity policies.

The introduction of economic instruments into environmental policy continues to be challenging. New levies with an incentive character regularly fail when goals are not specified clearly enough or when negative impacts for households with low and medium incomes are not neutralized:

- A people’s initiative for "internalization of external costs of energy" failed in a referendum with a record 92 percent of No-votes (2015). The initiative tried to replace existing VAT with taxes on non-renewable energies. Long-term fiscal distributional impacts were not transparent.
- A constitutional rule for an energy incentive levy launched by the Swiss government failed across all party lines (2017) and was abandoned. It was not clear what purpose the tax should serve. Renewable energy was supposed to be taxed while exemptions were provided for non-renewable vehicle fuels (EFV 2015).

As does the rest of the world, Switzerland is profiting from the benefits of the German “Energiewende” since renewable energies are getting ever cheaper. The Swiss energy transition lags behind Germany. It may only be a question of time before non-renewable energies will disappear because they cannot compete with power sourcing from renewable energies.

Regarding other carbon intense sectors—such as cement, air travel or waste recycling, technical solutions will be easier to introduce due to cheap clean power from renewable energy, and most countries are starting to use green hydrogen as a substitute for fossil fuels.

### New milestones coming with the Swiss CO_2_ law overhaul

A completely revised CO_2_ law aims to halve greenhouse gas emissions by 2030 from levels in 1990. However, a large portion of reductions is still perceived to be provided from “compensations” abroad and the amount of real “domestic reductions” remains controversial.

For the transport sector, carbon regulations for new vehicles will be tightened toward EU levels. Emissions from road transport fuels must be “compensated”; this may increase diesel and gasoline charges by a maximum of CHF 0.12 per liter by 2025.

For buildings, the CO_2_ tax on combustibles for heating can be increased to CHF 210/t of CO_2_. For old buildings, an annual CO_2_ limit per square meter of living space will apply when heating systems have to be replaced.

For industry, the linking of the Swiss emissions trading system (ETS) with the EU ETS has been achieved. The increased 2030 climate policy ambitions of the EU may affect Switzerland later. Large scale industries, air traffic and fossil-thermal power plants are included in the EU emissions trading system, but effective charges are not clear yet. Small, medium and large companies outside of the ETS can continue to be exempted from the CO_2_ tax if they agree to individual CO_2_ reductions.

No direct measures have been adopted against climate-damaging investments or involvements of the financial sector. However, the Swiss Financial Market Authority (Finma) and the Swiss Central Bank (National bank) have to review the micro- and macro-prudential financial risks of climate change.

For air travel, a levy of 30 to 120 francs will be imposed on plane tickets. A minimum 51 percent of the revenue from this is to be refunded to the population via the existing “carbon dividend” while up to 49 percent will go to a new “climate fund.” The new fund will provide financial assistance for new purposes including "appropriate research and innovation support, particularly in the aviation sector," financing the development of “renewable aviation fuel,” and measures will be taken to "stimulate liquidity bottlenecks by securing and standardizing energy contracting solutions for smaller buildings." Support should also be given to "hedge against the long-term risks of investments in the climate-friendly modernization of buildings, [electric] charging infrastructures in multi-party buildings, facilities for the production of renewable gases.”

If adopted, the new CO_2_ law will bring improvements. Still, loopholes may persist, and a large number of critical questions regarding implementation and enforcement remain.

## Priceless environment

### Neoclassical theory and environmental policy

Resistance to environmental regulation has some of its roots in neoclassical economics. Neoliberalism—a theory referring to market-oriented policies such as eliminating controls, deregulating capital markets, lowering trade barriers and reducing state influence—tends to ignore the environmental risks of economic activities. This resistance has its origins in economic theory which touts reductionist concepts of value.

The etymological root of “economics” describes the science of the "laws of the house" (ancient Greek οἶκος and νόμος). It can be interpreted as "budget management," i.e., dealing with scarcity, or as "housekeeping," i.e., conserving assets or values. In terms of proper semantics, economic science would appear to be predestined for the careful handling of natural goods. However, reality is somewhat different.

Economists tend to interfere in political discussions. But many of them remain surprisingly silent about the devaluation of natural and personal assets through climate change or through activities that maximize private benefits to the detriment of others. Their prevailing opinion is that markets are the best way to solve the problems of mankind. They regularly recommend government restraint as the best recipe for prosperity. This message is welcomed by the industries responsible for environmental degradation. Environmental protection is then left as the "personal responsibility" of entrepreneurs who have little room to act when exposed to competitive markets. Negative externalities will not be corrected this way.

### The promise of win–win solutions

The weakness of environmental and climate policy in recent decades has led to the emergence of many private and public initiatives in favor of *sustainable business* and *green investments* (PRI 2017). Companies and funds have been encouraged to review their business models according to ESG criteria. ESG stands for environmental [E], social [S] and governance [G] objectives for manufacturers, suppliers, investments, or funds. Economic activities should be analyzed and improved by reducing ecological footprints. Self-regulation is touted as a way for companies to defuse the antagonism between business and the environment (Austin 2019).

ESG efforts have been neither unsuccessful nor wrong. Innovative companies and committed consumers have made a huge difference. Quality labels for social and ecological value chains have sharpened the perspective on alternatives and realized benefits in many markets. Quality badges and innovations have been launched, and improvements have been welcomed.

On the other hand, we find that self-regulation and voluntary efforts have not been successful enough. In 2018 global CO_2_-emissions were 49 percent higher than 1997 levels, the year of the Kyoto Conference (BP 2019). Nation states still grant tax breaks, financial aid, and subsidies to extractive industries. Unsustainable fossil fuel value chains have prevailed, many of them heavily subsidized until now, such as the new liquid natural gas terminals in the European Union (Artelys 2020). Self-regulation has its limits when competitive alternatives are not on hand. Without sanctions, damage to the environment will increase.

The substantial profit levels that many oil and gas companies have enjoyed has caused continuous and successful lobbying against climate policy restrictions. Stakeholders of fossil (and nuclear) energy are experts in influencing press, television channels and parliaments. Since the UN World Conference in Rio (1992), too many initiatives have failed due to opposition from oil companies or countries that export oil and gas. For a long time, oil companies successfully denied the role of greenhouse gases and fossil fuels in global warming. Courts failed to hold the responsible persons and corporations accountable. Governments elected to remove regulations rather than to strengthen them; donations from extractive industries frequently helped pro-industry forces gain majorities.

Today, it is not knowledge that is lacking, but rather, appreciation for environmental values, goods, and services. And economic science shares responsibility for this (Schelbert 1991).

### Priceless: worthless?

Since the upswing of the postwar period, economic theory has assessed "values" and "value creation" using the method of calculating gross domestic product (GDP). Based on registered prices and quantities, the level of market transactions is measured as an indicator for prosperity. It is not the value of "usefulness" or the "existence value" of goods and services that determines prices, but rather the opposite: prices define economic values and people tend to believe recorded numbers.

Natural resources such as clean air, biodiversity and a stable climate are not traded on markets. They have *no price*. And hence they are routinely perceived to have *no value*. Natural capital does not appear in the balance sheets of companies or states. Maintenance of the integrity of natural capital is not included in the market equilibrium described as the Pareto optimum, the magic place where the "invisible hand of the market" has matched supply and demand so that no additional transaction can improve overall benefits.

Mass media communicate the transaction prices of companies (stock prices), commodities or exchange rates daily. However, you will rarely hear about value changes of natural capital or natural resources in the news; losses of natural assets are classified, if at all, as accidents, as when landslides destroy settlements, when countries fall victim to droughts or when animal and plant species disappear. All this comes at a cost, but from an accounting point of view it is done free of charge. Instead of talking about the pollution or polluters that are responsible, there is talk about “natural disasters,” as if earthquakes had started to dominate weather patterns.

In the former Soviet Union, there was a joke about the success of a planned economy: "The statistics were always good." But the queues in front of retail stores showed another reality.

Industrial societies have celebrated growth figures for decades. There is nothing wrong with this except that they obscure the extent to which our natural resources have been degraded and the additional risks we run over time. The climate crisis is endangering everything, and we can barely comprehend the extent and dynamics of environmental destruction.

In real economic terms, climate protection has not achieved the status of a reportable issue on a micro-economic level. Sustainability reports from companies are voluntary. Where they exist, they do not create enforceable obligations. And where avoidance technologies are not readily available or competitive—due to technical hurdles or high prices—environmental goals risk remaining unreachable.

### Value as a question of contemporary thinking

The Italian American economist Mariana Mazzucato points out that theories of value have always been ideological, and that they have changed over time (Mazzucato 2019). In *The Wealth of Nations*, Adam Smith made a distinction between productive activities that generate added value and unproductive activities, such as rent collected by landowners. “Value” was derived from the amount of work that went into production, first from agriculture (physiocrats), and later from industrial manufacturing (classics).

The school of marginal utility in neoclassical theory (from 1870) postulated that "value" was derived from marginal utility, the benefit gained from consuming one additional unit of a product or service. Though the usefulness of water may be great, the marginal cost is low and therefore its price is low or possibly zero. In the case of diamonds, as the supply is small, marginal cost tends to be high, though the total usefulness of diamonds in daily life is small (Lokanathan 2018). The school of marginal utility considers demand to be the decisive element in the determination of value. Its approach is from a micro-economic rather than from a macro-economic perspective. This myopic perspective often seems to dominate the thinking of many economists. Natural resources are priced using hedonic methods, by asking people what price they are willing to pay for a forest, for a clean river or for “climate stability.” *Value* thereby arises in the eye of the respective beholder, who feels free to give natural goods the value he wants from his individual perspective.

### The "tragedy of the commons"

The perception of value was different in the past. In the Middle Ages and well into modern times, important environmental goods—forests, fields and waters rich in fish—were managed as common-pool resources and local rules defined sustainable management (Ostrom 1999). The commons were protected against excessive use and the input of harmful substances usually remained unproblematic (Sieferle 1998). The hunters and gatherers of Stone Age, the first farmers of the Neolithic Age and the first urban societies cultivated animistic relationships with trees, waters, and landscapes. Natural goods were considered "sacred."

This changed with the expansion of market economies. The private acquisition of formerly common goods found new outlets and became an important source of wealth. Production was no longer a means of subsistence. The prospect of profit arose and the creation and extension of financial assets was made possible by the allocation of investments. A race for the appropriation of natural resources began, plundering virgin forests for rare timbers, oceans for whaling and fishing or clean air for fossil energy conversion to maximize monetary yields.

Natural resources, land, and entire continents were forcibly taken away from indigenous peoples and dedicated to the extraction of raw materials or the production of feed and food. Colonial structures encouraged corruption, violence, deforestation, and the depletion of soils and groundwater.

Whether or not humanity will survive these ruinous degradations is typically not a subject of economic analysis. The damage costs are borne by third parties, diffuse and often only show up as a problem over time. Market economics are based on the right to freedom. Today, everything that is not forbidden remains allowed. Shareholder value thinking is not focused on the preferences of future generations. The latter will have no choice but to accept what remains. Hardin (1968) described the consequences of this logic for common goods. If the pressure of use increases and there is no social control, everyone will maximize his or her yield even faster to maximize his or her current profit. Over time, the losses fall to the community.

Without values of cooperation or solidarity, individual rational conduct, as postulated by neoliberal-minded economists, leads to ruin. Methodical individualism, with its resistance toward state activity, did not oppose free riding behavior effectively. Common goods are neither inventoried nor managed by modern economists. Cheating and dining-and-dashing have become the main business model when it comes to natural goods. This will only change when environmental risks and pollution start to be sanctioned and when polluters are forced into avoidance activities and paying compensations that can maintain and restore natural capital.

### Correcting "market failures”: the deficits of Pigou’s theory

The British welfare economist Arthur Cecil Pigou (1877–1959) was the first to demand that not only monetary values but also all activities that generated “welfare” should be recognized as an addition of “value.” The role of the state would be to correct *market failures* when *negative externalities* caused damage but were not considered to be costs by companies.

Pigou's theory bears more questions than answers. Despite his broader perspective, Pigou remained a scholar of the neoclassical school of marginal utility. When extending welfare to include the environment, he had no macroview of natural ecosystems and the services they delivered.

### The "value of a forest”

The reductionist value perception of welfare economics can be illustrated by the concept of forest valuation (Altwegg 1988). There are several different “economic values” of a forest:

- *Value* = *Net present value (NPV)* The value of a forest is derived from the capitalized profit from the sale of wood (capitalized earnings value). Revenues are discounted. Long-term financial earnings count for less, while short-term profits count for more. The *conservation of forests* in the sense of sustainable management is not provided for. If meat production yields a higher NPV than growing timber does, priority is given to grass-based land use and the forest must give way.
- *Value* = *avoided damage costs* This method goes beyond private appropriation and takes welfare theory into account. It asks how high the public costs will be if a forest is destroyed. Estimation of damage costs bears uncertainty. The causality of implications and costs may be disputed (*denial*). When impacts become apparent only after time, the previously attributed value may prove to be too low and restoration will be difficult or impossible to enforce.
- *Value* = *avoidance costs* The value of a forest is calculated by the approved costs for substitutes that would be necessary to replace the former public service functions of a forest, such as protection against avalanches, landslides, torrents, rockfall or the drying up of soil. Preserving a forest may appear to be the cheapest option for maintaining its protective functions.
- *Value* = *willingness to pay* By this hedonic method, the value is estimated by investigating individual preferences. A target group, for example, inhabitants of a district, is asked how much they are willing to pay for preserving a forest. Awareness, income and the wealth of respondents will influence the results.
- *Value* = *willingness to accept* Here, the value question is given by asking people how much financial compensation they would need to accept clearing of a forest. Here, property rights are attributed to the public. It is not the conservation but the destruction of forests that is due for payment. This can lead to higher valuation, but rent-seeking is also not excluded when polluters (forest clearing companies) and recipients of compensation agree to deforestation.

### The missing dimension

With welfare economic concepts, despite their much broader valuation, the conservation of forests is by no means guaranteed. Sustainability is not a given when left to the question of preferences. The *first come—first serve* principle applies.

When environmental impacts are allocated asymmetrically between today’s generations and those born later, welfare economics and neoclassical theory tend to become a science of expropriation. Instead of safeguarding value, value destruction becomes a common habit and subsequent generations will find themselves cheated. They cannot successfully speak out against expropriation, and they are not part of decision-making processes. When irreversible effects on natural resources prevail, these values will be lost forever. To prevent this, the intrinsic value, or the "mere existence" of natural goods should be honored over time and safeguarding natural capital should be the rule, beyond individual preferences.

Pigou's concept ultimately leads to the *economically optimized destruction of nature*, where the preservation of natural capital might be an option but is in no way organized or affirmed as a common goal. Sustainability is not considered essential; therefore, natural capital is not protected (Söderbaum 2007).

How can sustainability be implemented in real terms?

### Making avoidance affordable: since damage costs are not

As we have seen from the given examples, organizing and financing environmental protection is possible and learning curves can be triggered. Environmental damage can be contained or undone by democratic movements, legislation and incentives. Market economies have always been creative. Today, they provide an unprecedented amount of private wealth in goods and services. So why should we not be able to recover public wealth through climate protection and other environmental protection measures, such as “net zero” emissions and a circular economy?

Division of labor, technical progress and mass production have improved productivity since the beginning of the Industrial Revolution. The efficient use of materials and resources was stimulated by innovation and competition. Today we produce more with less thanks to creative private entrepreneurship and checks and balances by states or nations. However, there are some caveats:

- Productivity gains were used far too little to reduce critical material flows such as nonrenewable energies or other extracted minerals. Instead of reducing them, they increased them (*rebound*), according to the motto: "My car uses less petrol, therefore I will drive more miles and abandon train trips".
- Market economies deliver "efficiency" only where production factors bear costs. Natural resources were too often taken for free. Accordingly, consumption increased without consideration of natural limits.
- Societies and states that can enforce standards and avoidance activities, for example, for sanitary measures in the handling of food or pandemics, have failed to protect global environmental goods for too long.
- Too often when environmental damage occurs outside of national borders, there is no regulation. Free trade agreements were door openers for destructive behavior and often acted as fire accelerators.

The politics of the nineteenth and early twentieth century were driven by a social quest. Children "should live better" than their parents, and indeed, they did; many indicators bear witness to this including income levels, increase in life expectancy, education and rest periods (holidays, third age, reductions in working hours). Prosperity grew. From the beginning of the Industrial Revolution, new infrastructure and inventions inherited by younger generations brought a rising level of prosperity. Today, however, this level is fundamentally threatened by a lack of respect for natural resources and their limits.

### Lessons learned from the German energy transition

In 1990, the re-unified German nation recorded high levels of fossil fuel consumption and the political debate on nuclear energy was unresolved. No independent observer was keen to give renewable energies a chance to grow exponentially and to conquer the power market back then. The electricity industry was closely interwoven with stakeholders from states, cities and communities. Many thousands of well-organized jobs were at stake when considering the phase out of nuclear or fossil-based energy.

The potential scale of renewable energy was underestimated even by its advocates, who emphasized the importance of *efficiency as an energy resource* while neglecting rebound effects as a minor problem. Renewables were still considered the more expensive option (1995).

The vision of full energy provision from renewables was categorically refused by the conventional energy industry—Hermann Scheer called this the *renewable energy lie* (Scheer 2010)*.* To be taken seriously in public, optimism about renewables was not recommended. Subsidies were, however, widespread–that is, for the old energy industry!

For coal there was the German "coal penny" until 1995. Nuclear power plants were legally exempted from paying for liability insurance and claimed to be profitable. Even later, when carbon emissions trading in the European Union was established, the environmental incentives were cancelled out by cost-free allowances for the main polluters. The energy industry was not supposed to incur costs for environmental damages. The sector was considered "strategic" regarding international competitiveness and low-income consumers. A political majority was willing to tolerate any external costs to keep energy cheap.

### The new policy

In 1998, the red-green government of Gerhard Schröder won a majority in the Bundestag and broke new ground focusing on innovation, including partial cooperation with the industry:

1. The core of the transformation was a price guarantee for all electricity from new renewable energy installations. The ambition was focused on building a diversified, competitive new energy industry, or in proper terms a *pollution avoidance industry*.
2. The nuclear phase-out was agreed based upon negotiations with the nuclear industry. Remaining operational lifetimes were fixed by law and perceived as a "compensation" to the operators rather than being faced with immediate closure. The risks of continued operation for some plants would be tolerated until 2022.
3. No “damage taxes” were imposed on nuclear and coal-fired power plants according to Pigou's allocative theory. The only new charge was the renewable energy levy (EEG-surcharge), which initially amounted to just €0.004/kWh. It later rose to €0.0676 €C/kWh, when the prices of the electricity exchange (marginal costs) started to be the base price for difference instead of the regular procurement costs of the power sector (Nestle et al. 2012)
4. The ambition to minimize social and economic casualties from structural change or ecological policy was a recurrent ambition. Energy-intensive companies (and others) were exempted from the EEG surcharge. Later, conversion costs of coal companies and nuclear waste costs were also socialized by the Angela Merkel-led coalition.
5. New technologies led to the decentralization of energy systems. This was fiercely supported by a citizens' movement, including ecologists, farmers, and traditionally conservative regions. The relocation of power generation to rural areas increased tax receipts of small municipalities and kept local businesses alive. The old, oligopolistic industry could not fight back.
6. Community wind farms, community solar parks and solar PV for self-consumption and storage created new, unprecedented business models. New opportunities were received with interest by capital market actors.
7. The regime of legally secured remuneration was a driving force for groundbreaking innovations. Cost reductions were achieved faster than expected. This massively helped the integration of renewables into conventional power markets and increased political acceptance. Competition and low marginal costs of power forced all market participants to learn and to adapt.

The energy transition was a driving force for German exports and for supplier industries, including many companies in neighboring countries. German and European industries benefited for decades, and Asian and a few American countries followed suit. Around the world, coal, natural gas, oil and nuclear power came under pressure. By spring 2020, Bloomberg New Energy Finance, a leading market analyst, summed it up: *"Solar PV and onshore wind are now the cheapest sources of new-build generation for at least two-thirds of the global population*” (BNEF 2020).

### Pigou’s internalization second, avoidance first

The case studies from Germany and Switzerland show environmental policy to be successful when avoidance technologies are introduced by an open, competitive, long-term approach.

Where polluting companies are open to prevention strategies and pollution avoidance, cooperation amongst consumers, producers and local authorities makes sense. However, there is the risk that companies cooperate by abusing their knowledge to water-down new standards and goals. For governments, it is crucial to have independent experts and knowledge about *best practices* and innovative technology.

In cases of technological stalemate, it is crucial that companies do not bear avoidance costs single-handedly. New technology should then be funded by industry-wide cost allocation such as the waste charges or heavy goods transport levies in Switzerland or the EEG-levy in Germany.

In Germany, substantial efforts were carried out by research institutions, citizens, new companies, small investors, and farmers. For too long, incumbent companies waited at the sidelines and started to get involved only when super-sized investments such as those for offshore wind were asked for.

### The role of government

Usually, several technical solutions are identified to avoid environmental problems. The crucial question is who acts and who bears the rather high short-term avoidance costs. This is exactly the moment when governments can help. First, they should formulate environmental goals in physical dimensions such as emission or waste volume over time.

Then, they should manage the variety of different solutions, eliciting new avoidance strategies and testing old ones by designing a comprehensive transition path including educative and social arrangements for companies that are left behind.

The search for avoidance technologies should be open and should include competitive elements. The role of government is, indeed, multiple:

- Defining environmental goals (emissions, critical levels, critical loads)
- Funding research and development
- Enabling pilot plants and pilot processes
- Clarifying locational issues and permitting privileges
- Quality control and documentation of progress
- Financial support for market introduction
- Introduction of avoidance/abatement charges including sector-wide "pay-as-you-go" schemes
- Adaptation, social facilitation, damage compensation
- Introducing a “Pigou tax” on remaining emissions.

### Stages of change

In organic and "fair trade" farming, it was private individuals who created markets for new value chains with ESG-profiles out of their own pockets. In forest protection, freight transport and energy policy, damage prevention was achieved by changing property rights even while polluters fiercely resisted. Later, however, the newly established regulations and technologies enjoyed a high level of acceptance among former opponents, turning them into big investors.

With market- or sector-based avoidance strategies, the discussion on “Pigou taxes” should be replaced by a discussion of product, sourcing or processing qualities. When, around 2015, power production from renewable energy reached cost parity with new coal and gas plants, German, French, English, Spanish, Norwegian, Portuguese and Italian utilities and power companies such as (in alphabetical order), BKW, EDF, EDP, EnBW, ENEL, Engie, E.on, Iberdrola, RWE, SSE, Statkraft and Vattenfall all started to reposition themselves in the new power markets (Amelang et al. 2016; Brunekreft et al. 2016), just to name some important European actors. Their investments in renewables often started in new, far-away places outside the established supply areas. This changed over time, and residual conventional power plants “at home” were replaced, split from the main company, or transferred to a “bad bank holding” that would later be sold.

In the long run, cost allocations for avoidance should comprise all levels of the value chain including cross-border trade flows, for example for wood, palm oil, cocoa or cotton. Intergovernmental trade agreements should ensure that ecological production can be established by itself for a commodity’s quality, and quality assurance should be an obligation for the overall value chain. In the EU, this was made possible when requests for proposals for renewable energy tenders were organized to include trans-border areas.

The necessity of internalizing the remaining external costs of harmful goods or services that are not avoided will persist. Harmful goods or behavior should never remain "cheap." This also applies to residual emissions of so-called better products or “avoidance industries.”

The timing and amount of avoidance cost surcharges are delicate decisions. Once new techniques are available, new charges are more easily applied, and they may turn out to be a boost for the industry when overall cost in the sector can be reduced, as has happened with new renewable energy investments.

When avoidance costs continue to be expensive, the preference should be for reducing the cost of better products while substituting market shares of the old, polluting practice. As soon as low-emission alternatives are available for a reasonable price, an additional charge on externalities “Pigou Style” can be an option, amongst others. Often a regulatory ban, a final closure period or an emission standard can do the same when the avoidance of harmful products and processes has become common sense. Internalization charges can wait, but they must remain a credible threat if a sector does not improve.

If a Pigou tax had been introduced in Germany in 1990 as a starter, it would have triggered only minor impacts while creating competitive disadvantages for export industries and drawing fierce political resistance. Power companies would have passed the new levy onto consumers, remaining largely unscathed, and the latter would have felt confirmed in their prejudice that insatiable governments always find excuses to charge innocent citizens. The expansion of renewable energies probably would have failed.

### Lacking acceptance for pure incentive levies

In early stages of the deployment of a new avoidance strategy, partial support from state budgets for research and market introduction do not necessarily reduce efficiency. The primary aim is to create consensus for change through new technologies or processes. A step-by-step micro-start in niche markets with modest costs, as the EEG has done, seems like a good option.

It is no coincidence that today, coal-fired power plants are stigmatized while being subject to new EU-wide carbon regulations. However, this happens only now, when renewable energies have achieved a competitive position. Thirty years ago, moral shaming and blaming would hardly have been successful. When avoidance technologies are close to being competitive, it is the right time for the internalization of external costs. In 2017, the EU emissions trading system was supplemented with a market stabilization reserve, lifting the price of CO_2_ certificates, while incumbent companies with new investments and old allowances took a profit.

From 2018, an increase in CO_2_ emission costs to > 20 €/t CO_2_ across the European electricity market was registered. This time, despite being a homeopathic dose, the still rather low price for carbon emissions turned out to be the most effective climate protection measure of the last 20 years over the whole European common power market. It made coal-fired power plants unprofitable, while cheaper energy from new renewable investments was ready and available. The price of avoiding technologies had fallen so far that a difference of € 0.01–0.02/kWh was sufficient to make a change.

### Dynamic efficiency of policy instruments

However, when looking at avoidance technologies, the dynamism of environmental policy instruments differs sharply.

*Rules and standards* do not aim for a change in product prices by accounting for externalities. By imposing emission limits on products, *basic standards of avoidance* must be achieved by polluters. Depending on the strictness of standards, avoidance costs add to the final price of products. Standards mostly work gradually. *Residual emissions or externalities are not subject to charges*. Therefore, incentives for avoidance innovations are modest since there is no incentive to do more. Moreover, governments are reluctant to implement sharp standards or levies when imported products or services are left un-charged.

*Subsidies and tax reductions *can deliver economic incentives to protect the environment. But they come at a cost while leaving the polluter-pays-principle aside. They may cheapen a perceived "good" product and sometimes accelerate innovation—for example, with public transport, organic farming practices or with tax deductions for renewable energy output. *Subsidies* may reduce emissions and environmental damage. However, the effects of incentives are limited if governments still fail to charge polluting products for externalities. Moreover, support for innovation may include a considerable regulatory risk. State budgets are limited; subsidies and minimum compensation rules may disappear when majorities change. When feed-in-tariff systems in Europe were altered retroactively in Spain, Czechia or Switzerland, investors were hampered.

*Levies, eco-taxes, liability provisions, emissions trading and combined incentives *can be considered as preferable PPP-solutions for environmental policy. What is crucial for the impact of these instruments is the combined result of a price incentive for internalization and spending revenues.

Support of renewable energy from *emissions trading* has been rather weak. For many years, emission trading systems of the EU ETS were insufficient in creating stable financial support for clean energy investments due to low CO_2_ emission prices, free access for allowances and volatility. This gradually changed for the better when a market stability reserve (MSR) was introduced to reduce the volume of authorized emission permits, giving carbon emissions a kind of minimum price similar to the UK nominal carbon minimum price introduced in 2013.

Levies are difficult to introduce, but they may be particularly innovative when designed as “feebates" (Hawken 1999), a self-financing system of fees and rebates that are used to shift the costs of externalities onto the responsible market actors.

Feed-in tariffs and recycling fees work similarly: A small fee on harmful products is used to finance avoidance activities, ideally initiating learning curves and cost reductions. Successful examples are the German Renewable Energy Act (EEG) or the Swiss waste guidance where learning curves have been initiated, enabling steep cost reductions for new technologies.

While at the start, cost-plus regimes for avoidance activities may open new doors for a high number of different options, avoidance activities, financed by allocated fees, should be organized in a transparent, competitive manner when established as “best practice.” In the long run, avoidance activities can be auctioned to enhance cost reductions, as observed with renewable energy.

### No tax: an apportioned fee

The German feed-in-law (EEG) did not rely on subsidies for investments nor did it make payments to individual manufacturing companies. Instead, it relied on a cost apportionment (“apportioned fee”) which was allowed by law to be passed from the buyers of power, mainly utilities, to the final consumers of power. This mechanism had many advantages:

1. Tariffs were not paid for new capacity (kW) but for the supply of clean electricity (kWh). There was low risk for "white elephants" to be bred by investment subsidies, as happened in many nuclear power deployments.
2. Performance-related compensations for real production (kWh) fed to the grid also proved to be effective prevention against "picking winners." All suppliers had the same access to market.
3. Citizens, farmers and investment funds were on equal footing with regard to feed-in tariffs. The diversity of actors improved public acceptance.
4. Financing by apportioned, cost-based sector surcharges protected producers against fiscal budget cuts. The power sector had to cure itself from within.
5. Thanks to the apportioned fee, services were *protected against the non-affectation principle* that is widely practiced in budgetary policy. According to this principle, the revenue side of a public household is separated from the expenditure side. This means that there should be no direct connection between how money is collected by governments and what purpose it serves. The prohibition of earmarking is meant to protect legal decision makers from making the same decisions as their predecessors. While this principle is valuable in fiscal policy, in environmental policy it is not. For the latter, the polluter-pays principle is regarded as the preferred system of cost allocation. This should account for damages done, damages undone and damages avoided. Here, it makes no sense to dissolve fees from their purpose or cause.

The apportioned fee system was particularly important for ensuring legal support by the European Union (EU) legal bodies. Feed-in tariffs were therefore not assessed as "aid," but as a minimum remuneration for a special quality of product. The lack of direct payments from state budgets made legal acceptance possible. Years later, the EU recognized the high effectiveness of feed-in tariffs: *"Well-adapted feed-in tariff regimes are generally the most efficient and effective support schemes for promoting renewable electricity"* (European Commission 2008a).

Another important finding was that there was no need to wait for global climate agreements or harmonized carbon taxes in order to develop abatement or avoidance activities. A single, medium-sized country (Germany) together with a number of partners managed the switch to new, cleaner energy sources almost single-handedly. This environmental achievement was in line with EU values, and compulsory targets for renewables for all member states were set in 2009 (European Commission 2008b).

### Environmental policy must be on its guard

Economic science provides a canon of apologetic theories opposing government intervention (Klink 1994). According to the author of public choice theory, James Buchanan (1919–2013), *state failure* is more problematic than *market failure* because so-called bureaucrats will make the situation worse.

The concept of state failure consists of a hodge-podge of ideas where political decision-makers are primarily concerned with personal gain. They will damage the common good through nepotism, cronyism and corruption. According to this view, environmental protection merely leads to wasting public money on unsuccessful technologies that crowd out private investment (Tullock et al. 2002).

This kind of thinking became a popular movement in the USA, boosted by billionaires who pursued their own interests. The *Tea Party* conquered the Republican Party and led to the candidacy of Donald Trump ("America first"). In some cases, governments and reform-oriented parties are also to be blamed for the recent surge of populist parties. For too long, governments have ignored income disparity and have lost trust and credibility. In France, the *Gilets jaunes* opposed and prevented CO_2_ levies because the Macron government was unwilling to compensate for the additional burden of any kind of “carbon dividend” such as the one in Switzerland that neutralized the reduced purchasing power of a CO_2_ levy.

Environmental policy must therefore be on its guard. Benefits of government intervention must outweigh the costs (Buchanan 2003). This can be achieved by a gradual start, testing and cooperation with established companies who are willing to change. Actors in the creative private sector are normally intrinsically interested in new technology and innovations. The ESG movement for responsible investment is a good example.

When technology partnerships can develop, environmental policy will find its stakeholders in cross-party-alliances. This is an important element in gaining public acceptance.

## Final thoughts on clean flying

Climate research predicts an impending rise in global temperatures and delayed, nonlinear (explosive) costs (IPCC 2018). The increase in environmental degradation exhibits patterns of an epidemic. Time is an important factor. Failure to act will be ever more expensive, and tipping points must be avoided as far and as quickly as possible (Sachverständigenrat 2019).

Over the last 100 years, temperatures worldwide have risen by 1 degree Celsius. So far, CO_2_ emissions have not been stabilized. As a result, temperatures have started to rise even faster—by an estimated 0.2 degrees per decade (IPCC 2018). Even with some CO_2_ reductions in the medium term, we will be far from reaching climate stability; damages will continue to grow. Therefore, achieving “net zero” emissions should be the way forward.

It is a battle on two fronts: The cost of destruction is increasing. And there are transition costs for a “low-carbon economy” that increase prices for end consumers. But is this true? Following learning curves, we can view this transition as an opportunity, too. Avoidance investments within a "Green New Deal" will reduce direct energy costs, while in some sectors such as airline industries rising costs might be unavoidable. Repair and “healing” costs could create new business cycles, not necessarily increasing welfare but stabilizing the climate, and therefore avoid disaster. Defensive activities are necessary to escape the worst anticipated damages.

**Fig. 14:**
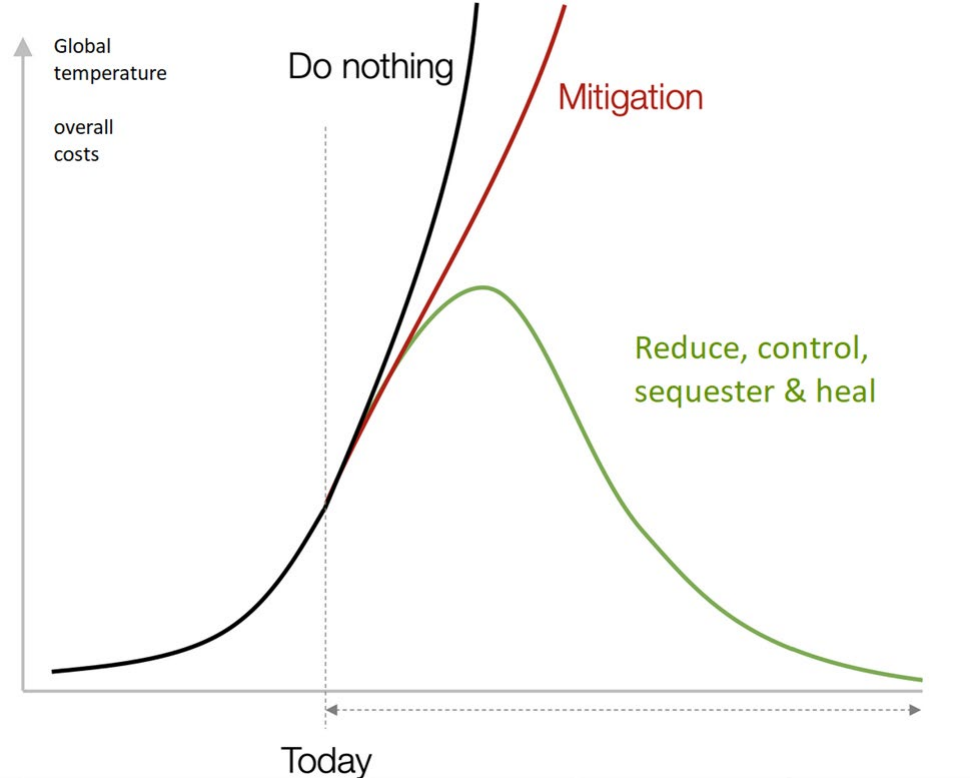
Climate policy paths (graph by the author, inspired by Pueyo (2020)

A comparison with the reactions during the COVID-19 epidemic is of limited benefit (Fig. 14). Nationwide measures will not lead to a corresponding slowdown in global warming but may bring indirect successes. The example of the EEG shows that national efforts or a *coalition of the willing* can make expensive technologies cheap and change international sourcing and trade patterns. The dissemination of low-carbon technologies has driven coal power plants out of markets where competitive electricity sourcing is the rule. Avoidance has become cheaper; hence, the move toward sanctioning polluting technologies on political grounds has become easier.

The latest photovoltaic and wind power technologies produce 100 times less CO_2_ emissions per kWh than power from lignite (Ballif 2020, 27; Wissenschaftlicher Dienst 2018). With growing market shares, multiplier effects can be expected and open new doors for a circular economy through the processing and recycling of materials that were previously considered waste.

### Moral escapism: a dead end

The climate crisis will not be resolved overnight. Where it is difficult to cut CO_2_-emissions and where technical solutions are not at hand, polluters normally exhibit reluctance toward change. One of the sacred cows is air traffic. For decades, political approaches to emissions management were insufficient or uncertain at best.

New terms such as "flight shame" are an indication that many people are unhappy with the situation. As a result of perceived helplessness, the debate has started to become moralized. People question air travelers who take advantage of cheap offers. And some passengers themselves feel *ashamed of flying*.

The Viennese ethicist Konrad Paul Liessmann gives some warnings against moralization. For instance, moralizing might block conflict resolution:

"For me, the question of what energy we use is primarily not a moral question, but a question of political concepts on energy, mobility, and industrial production. It is a question of weighing up and balancing different interests. By moralizing these questions, firstly, you distract from looking at things in a sober way. Moralizing always takes place in a mode of indignation, blame and shame. It clouds the view” (Liessmann in: Fuhs 2019).

Through moralization, social, technical or political challenges become a question of the misconduct of individuals. This can be a successful strategy if you want to *prevent* regulation. By allowing this, politics is cheated out of its proper tasks, while people start to believe that solutions are impossible.

"Then you do not need laws, but those who drive the wrong car model simply are evil. I do not think there is much to be gained if people keep flying but are ashamed to do so. This inevitably leads to rituals of shame and expiation, but no improvement in the carbon footprint" (Liessmann in: Fuhs 2019).

Moralization itself has a social component, both in patterns of consumption that we morally condemn and in those from which we cannot escape:

"Once it is the diesel engine, then the airplane, then the cruise ship, then every newborn baby. We exclude things that are important to us, anyway. Who talks about the devastating CO_2_ balance of digitalization? Nobody.[…] Moralization leads to a competition of expressions of a good conscience, and this good conscience, by the way, also has its price: one must be able to afford to live a climate-neutral life, or at least pretend to. Nothing is won" (Liessmann in: Fuhs 2019).

Individual changes of behavior might not be as insignificant as Liessmann believes. If somebody decides to travel without flying to protect the climate, it certainly is a contribution to climate protection on a micro-level. And micro-changes by thousands of people may create new value streams and macro-restructuring—for trains, for example—and be the start of different behavior patterns. In Europe, there are many people who have given up flying or reduced their air travel for climate-related reasons.

However, individual avoidance activities may not be sufficient in the fight for climate protection. Moreover, some strategies perceived as avoidance technologies might not be "good" enough based on life cycle assessment. Liessmann talks about these *grey zones*:

"It is possible that our energy-hungry technical civilization has maneuvered us into a situation where there are no longer any clearly positive solutions, where everything we can do will involve major risks and disadvantages. Moralization pretends that there is good and bad, and thus actually trivializes the situation. The individual can of course do something. He can try to move things along the paths of political decisions, can argue and launch new concepts to convince people. That is hard work. But that is the very essence of a democracy.[…].

Companies react to markets. You can create a gentle pressure on companies to influence corporate strategies. Customers can boycott certain products or change their buying behavior.[…] We can set a good example, but we must be very careful not to fall into the trap of double standards. It is a different matter when you come to a decision at a political level to change the legal frameworks […] in principle, be it regulatory or fiscal. Then I don't need any moral at all" (Fuhs 2019).

Too often though, in the face of relentless lobbying, “Pigou-style” internalization attempts and the discussion of damage costs have led to decades of deadlock. Instead, focusing on avoidance options might be more successful.

### Communication tasks

A main communicative task is the creation of empathy for the needs of the future and for the survival of younger generations. As global warming has progressed, the damages alleged to be “problems of the future” are spilling over into the present. Extreme temperatures and their costs can no longer be kept away from headlines. Insight is growing into how avoidance activities for later generations may bring benefits to those living today as well, by reducing local air pollutants, noise or preserving biodiversity.

With a consensus for action, experiences gained through the EEG should be adopted in other industries. This would mean that avoidance policies develop step by step:

- *Phase 1: Developing avoidance technologies* Begin on a small scale with establishing low-emission products and services with a minor market share, as has been done with renewables in the early years (before 2000) or with organic farming in agriculture.
- *Phase 2. Create long-term programs* Innovations with a smaller footprint should be assessed regarding their footprints and—if solid—get an assured remuneration within a fixed time frame, sponsored by industry-wide cost allocations (small, sector-wide contributions). Basic research should be financed by governments. These two financial flows were successful with renewable energies in Germany or with the recycling business in Switzerland. The philosophy behind it is that products and processes, including avoidance of environmental or social damage, should not be more expensive than their conventional counterparts.
- *Phase 3: Scaling up* Once avoidance technologies are established technically at reasonable costs, obsolete technologies will have to give way, incentivized by “feebates” (a mixture of small charges and rebates), emission trading, technical standards, decommissioning time limits, bans or—in a later stage—by incentive levies (Pigou taxes) combined with a “dividend” reimbursing substantial parts of the charges while preserving the social balance.
- *Phase 4: Internalization* When the change in technology has progressed successfully, environmental damage might still occur from new technologies with a better footprint, depending on the product or industry. For remaining damages, pollution charges, prevention and compensation schemes should be continued. It might be necessary to finance "negative emissions," for example, with the planting and burial of forests (Zeng et al. 2008) for bio-carbon capture and storage.

### “Clean Air traffic”: a draft

Let us outline a model of feed-in tariffs for the airline industry as an intellectual experiment:

1. *Developing avoidance technologies* Create an inventory of sustainable avoidance technologies for the technological field of air travel. Exclude polluting, extractive non-renewable energies, or problematic renewable energy sources (such as biomass from clearing forests or from farm-based biomass with high straining of rich natural terrains) from support; create a list of admissible technologies as eligible for support. Increase funding for research.
2. *Support of long-term programs* The government—or better yet, an alliance of governments—should then offer cost-covering minimum remuneration for “clean flights” over a predefined, long-term time frame, open to all companies and technologies that meet the requirements. The minimum price should be based on performance (e.g., distance) or prototype costs. The “new way to fly” should be offered to consumers at the same price as tickets for conventional flights. Financing should come from an industry-wide charge backed by legislation.
3. *Scaling up* Once new technologies are established at reasonable costs and with positive results, goals to reduce the amount of polluting transportation should be enforced by increasing the use of avoidance technologies incentivized by more “feebates,” a mixture of charges and rebates. For short distances, this could also mean that a rail system is boosted instead of air traffic, financed by flight passengers.
4. *Internalization* Residual amounts of pollutants should be taxed on equal footing with other climate policy instruments. Revenues should then be distributed as a “dividend,” for avoidance activities and for “negative emissions” along the lines of the Paris agreement.

In terms of product costs, learning curves should be considered.

### Outlining the example of a Zurich–London–Zurich air travel

A train ticket Zurich–London–Zurich has a price of € 670.—(2020). The journey takes 16 h. It is six times more expensive than a flight which costs € 105.—(2020) and takes about twice as much time (airport waiting periods included).

In economic terms, willingness to pay seems to be very high for flight services. Today, these trips are only marginally substituted by trains.

According to the logic of the “Energiewende,” all companies with sustainable *carbon–neutral aircraft* should receive a cost-covering tariff, be it some €700 instead of €105 per passenger, while clean flying passengers are able to pay the same ticket price as conventional passengers.

Higher compensation finances more avoidance activities over time. To be feasible, the whole scheme must be long term. A reassurance for investors against retroactive reduction of compensation may be crucial in initial stages. The price schemes for *clean planes* will be reduced as soon as new models get cheaper.

A “clean” Zurich–London–Zurich ticket may initially cost several times the current price; however, the market is huge and air passengers have a high propensity for flying. The additional *surcharge on all tickets* of a certain country may be modest when clean flying has gained a minor market share.

**Fig. 15:**
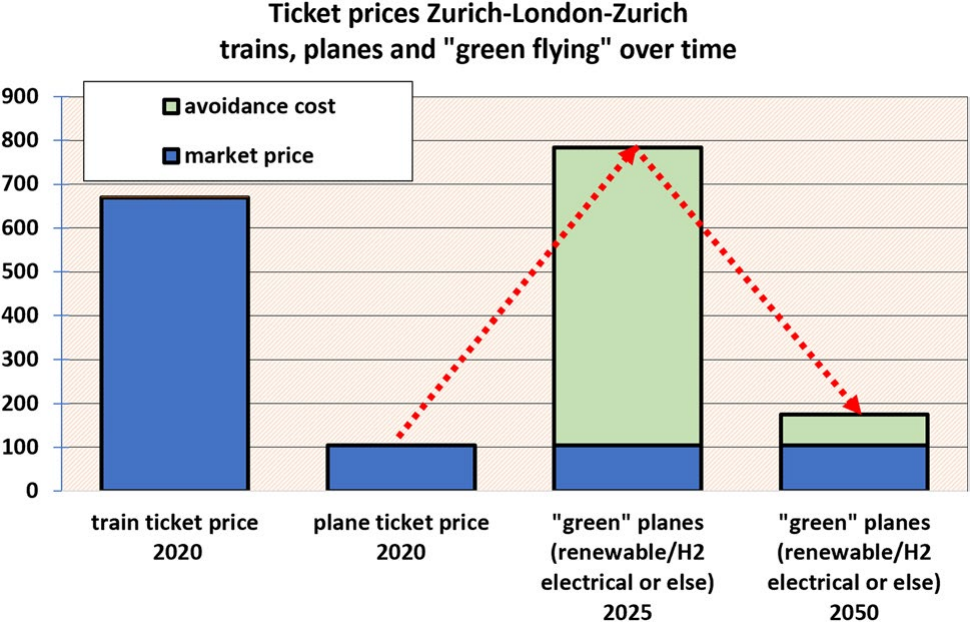
Market prices and avoidance costs of a trip Zurich–London–Zurich (by the author)

Over time, these costs are projected to fall due to the learning curve, and low-carbon air travel will get cheaper. Then, the market can be scaled up and costs for clean flying may be reduced again (Fig. 15).

The fact that governments are looking for innovative products is nothing new. During the Cold War, militaries had very large funds for defense. These budgets were powerful engines of innovation. Actors were rarely under pressure to justify their expenses. Doubts were considered "unpatriotic." The same logic may apply today if climate protection becomes as important as military defense.

## Conclusion

The German energy transition, including the persistence with which it has been achieved, is of similar relevance as the innovations in information technology in the Silicon Valley. What distinguishes it from private innovation is its quality as an avoidance industry that arose primarily through parliamentary initiatives and decisions. The courageous, cost-based compensation schemes decided by the German Bundestag were inspired by the idea of dynamic efficiency and by the polluter-pays-principle, operating completely outside of the normal budgetary rules for governmental support. They delivered the necessary long-term support while maintaining crucial incentives that are specific to economic instruments.

The decisive factor for success was not research but to a much larger extent, as the term *learning curve* suggests, market introduction, scaling of volume including priority access to the grid. With a variety of coalitions, the German Bundestag maintained these financial conditions for initially expensive “alternatives” over time, until they achieved a cost-effective, mainstream status and were able to conquer energy technology markets worldwide.

It was a stroke of luck that the actual trigger for this energy sector transformation was based on broad opposition against nuclear energy. Nuclear energy was politically battered in Germany after the catastrophe at Chernobyl. It has never achieved the strategic position it has in France or Great Britain, where it is part of military strategy. Nuclear power stations always had smaller market shares than coal-fired power stations in Germany. If the energy transition had been directed against the German coal complex from the outset, it might have failed due to political resistance long before renewable energy reached a competitive status.

The importance of this political constellation is particularly evident when compared with the pitiful failure of the German car industry, which was unable to transfer the achievements of clean power from renewable energy to the transport sector on its own. It only switched to a sustainable energy supply for vehicle production later, when it was under pressure from foreign competition and after fatal criminal machinations around diesel emissions and IT-malware in its cars.

From the German energy transition, we can learn that avoidance schemes can be highly successful and may be transferred into new sectors of the economy. For this to happen, the institutional backing of circular economy principles at a constitutional level is advantageous. Or at the very least, individual sector transitions should be supported by legislation based on PPP-deliberations, keeping long-term support systems outside of fiscal budgetary mechanisms.

Perhaps in the future, every economic sector or market will need institutional arrangements with supervisory duties similar to the “forestry service” of the Swiss Forestry Act. The task would be to.

- analyze sustainability gaps along the supply chain and consult science, industry and NGOs;
- provide sufficient funding for the market introduction of new technologies or processes that prevent externalities, at least in the initial innovation phase, and
- make financial resources available on the basis of an industry-wide cost allocational fee until these techniques have passed the learning curve, whereupon they become a least cost "best practice," as demonstrated by renewable energies.

Delegating the task of monitoring the supply chain to the individual consumers, as recommended by neoliberal economists, is not very helpful. This is because transparency at the end of the supply chain is often inadequate and ends up in a jumble of different labels, whether credible or not. Even well-intentioned consumers are often unable to judge the ecological footprint of a product and to influence a change in production methods, because responsibility ultimately lies with companies and because the entry costs for the latter are often too high to launch expensive innovations against competitors.

Self-regulation is certainly an ideal solution. The efforts and scope for action of ESG-oriented investment funds and ESG-oriented corporations should not be underestimated. However, there are sectors—first and foremost the suppliers of fossil and nuclear energy sources—that remain categorically opposed to reform decade after decade and where efforts to find alternatives within the sector are futile.

This means that the responsibility for solving environmental problems falls primarily back on political authorities. Whereas the economy does not in itself guarantee a circular flow of materials and sustainable production methods, it is they who can prescribe and finance this, and ESG-friendly companies and investors might join these efforts by launching partnerships with state agencies delegated to realize circular economy material flows.

The classic approach of the Pigou tax falls short in this respect. The aim must not be to tax damage, but to prevent damage by innovations and rearrangements of production and processes. To achieve this, it is important to find partners in the economy and to give them the opportunity to bring their products to market while shielding them against economic disadvantages.

In the case of renewable energy, this was guaranteed thanks to the resolutions of the German Bundestag, supported by a competent industry, committed research institutions in Germany and around the world and hundreds of thousands of citizens who dedicated themselves to the adventure of decentralized energy production long before it became a standard.

The German example also shows that the omnipresent cost argument against such new environmental economic instruments has, in reality, been overcome. In the foreseeable future, Germany will have not only the cleanest but also one of the most competitive energy industry, and the innovations and implications of the German energy revolution will open up new market opportunities and a variety of other possibilities for the production of circular material flows and the reduction of emissions and hazardous waste.

## Footnotes

1 Wind turbine model Kenetech KCS-56, built in 1985 and rated at 100 kW, had a rotor diameter of 17 m; in January 2019 S Gamesa presented offshore wind turbine SG 10.0-193 DD with a capacity of 10 MW and a rotor diameter of 193 m.

2 The former Risoe nuclear research center (Roskilde, Denmark), where research concentrated on wind turbines from 1985 onward, made major contributions; in Germany, the Fraunhofer-Institut für Solare Energiesysteme (ISE, Freiburg im Breisgau) and the Fraunhofer-Institut für Windenergiesysteme (IWES, Bremen, Hamburg, Hannover, Oldenburg) were outstanding centers of research; in Switzerland, the Universities for Applied Sciences (Burgdorf, Rapperswil), the Centre Suisse d' Electronique et de Microtechnique (CSEM, Neuchâtel), and the Swiss Polytechnic Institute (Lausanne and Zurich) were important along with many others in Japan, Australia, Spain, the US and China.

3 Nimby (Abbr.) “not in my backyard!”.
